# Anger Experience and Anger Expression Through Drawing in Schizophrenia: An fNIRS Study

**DOI:** 10.3389/fpsyg.2021.721148

**Published:** 2021-09-01

**Authors:** Wenhua Yan, Weidong Ji, Chen Su, Yunhan Yu, Xiaoman Yu, Liangliang Chen

**Affiliations:** ^1^Shanghai Key Laboratory of Mental Health and Psychological Crisis Intervention, School of Psychology and Cognitive Science, East China Normal University, Shanghai, China; ^2^Affiliate Mental Health Center, East China Normal University, Shanghai, China; ^3^Shanghai Changning Mental Health Center, Shanghai, China; ^4^The School of Psychology and Cognitive Science, East China Normal University, Shanghai, China

**Keywords:** schizophrenia, emotion experience, drawing, anger, fNIRS, emotion expression

## Abstract

Differences in emotion experience and emotion expression between patients with schizophrenia and the healthy population have long been the focus of research and clinical attention. However, few empirical studies have addressed this topic using art-making as a tool of emotion expression. This study explores the differences in brain mechanism during the process of expressing anger between patients with schizophrenia and healthy participants using pictographic psychological techniques. We used functional near-infrared spectroscopy to fully detect changes in frontal cortex activity among participants in two groups—schizophrenia and healthy—during the process of experiencing and expressing anger. The results showed that there were no differences in the experience of anger between the two groups. In the process of anger expression, the dorsolateral prefrontal cortex, frontal pole, and other regions showed significant negative activation among patients with schizophrenia, which was significantly different from that of the healthy group. There were significant differences between patients with schizophrenia and the healthy group in the drawing features, drawing contents, and the ability to describe the contents of their drawings. Moreover, the effect size of the latter was greater than those of the former two. In terms of emotion expression, the drawing data and brain activation data were significantly correlated in each group; however, the correlation patterns differed between groups.

## Introduction

### Emotion Experience and Emotion Expression in Schizophrenia

Schizophrenia is a mental illness that impairs mental and social functions. Its pathological characteristics include delusions, hallucinations, disorders of thought, language, and behavior, as well as negative symptoms (American Psychiatric Association, 2013). Negative symptoms include apathy, loss of motivation, and social withdrawal (Van Os and Kapur, 2009). Schizophrenia affects patients’ thoughts, cognition, language, feelings, and behavior (Schultz et al., 2007; Van Os and Kapur, 2009). As emotion plays an important role in social interactions (Kring and Moran, 2008), mood disorders can affect patients’ personal and interpersonal abilities as well as their social functions. Therefore, the problem of mood disorders in patients with schizophrenia has long been the focus of research (Kring and Caponigro, 2010).

Research on mood disorders in schizophrenia has focused primarily on emotion experience, emotion expression, and emotion recognition (Trémeau, 2006), of which this study focuses on the first two. In recent years, a number of meta-analyses have shown that compared to the normal population, patients with schizophrenia have no difficulty in experiencing “in-the-moment” emotions (Kring and Moran, 2008; Kring and Caponigro, 2010; Yan et al., 2012; Ni et al., 2017). However, a meta-analysis revealed that although patients with schizophrenia had no defects in experiencing pleasant emotions, they experienced increased levels of negative emotions (Cohen and Minor, 2010). Other studies have demonstrated that most patients with schizophrenia experience emotions similarly to healthy people, although some subtypes of patients differ in this regard (Strauss and Herbener, 2011; Oorschot et al., 2013).

Many studies have shown that patients with schizophrenia have disorders of emotion expression—a condition known as alexithymia. This causes problems in identifying and describing their emotional state and makes it difficult for individuals to identify, process, and regulate emotions (Kring and Moran, 2008; Ni et al., 2017). Emotion expression usually involves the following aspects: it is expressed through observable external mechanisms such as language, facial expression, posture, or behavior; is associated with internal emotion experiences; and tends to be associated with the social meanings they transmit (Zhu, 2013; Keltner et al., 2019; Olderbak et al., 2021). Research on alexithymia in patients with schizophrenia has focused on the first aspect, as expressed through facial (Berenbaum and Oltmanns, 1992; Aghevli et al., 2003; Gaebel and Wölwer, 2004) and vocal expressions (Levin et al., 1985; Alpert et al., 2002; Trémeau, 2006) in particular.

### Expressing Emotion Through Art-Making

Art-making not only allows expression of the inner experience of emotion but also enables individuals to give symbolic meaning to the works they create, thus making the art they generate socially significant. Therefore, art-making can be a useful tool for the study of emotion expression. Surprisingly, although drawing techniques in art-making are inherently self-expressive (King and Kaimal, 2019), and the process of drawing enables the creators to express their own emotions (Guseva, 2018; Reyna-Martinez et al., 2019), few studies have used the drawing task to explore the characteristics of emotion expression in patients with schizophrenia. Previous studies using drawing tasks have mainly used the clock drawing task and tree drawing test to explore cognitive function and motor ability in patients with schizophrenia (Bozikas et al., 2004). Moreover, the house-tree-person test has been used to study depression, anxiety, and other related mental health states in schizophrenia patients during the convalescent period (Ma et al., 2013). Many studies have also used art therapy to intervene and alleviate some symptoms in patients with schizophrenia (Gühne et al., 2012; Qiu et al., 2017; Lee et al., 2019; Tong et al., 2021). However, there are few empirical studies on the connection between drawing and its internal emotion experience, emotion expression, and the social meaning it transits, or how these are connected to brain nerves. This study focuses on identifying the differences in emotion expression through the drawing task between patients with schizophrenia and a healthy population using brain neurotechnology techniques. The expressive therapies continuum (ETC) model comprehensively and systematically explains the mechanisms underpinning information processing and image formation in interactions between individuals and media, combining information processing with the creation of art. It thus provides a framework for understanding artistic creation in different groups, including healthy people and patients with schizophrenia (Kagin and Lusebrink, 1978; Hinz, 2018; Lusebrink and Hinz, 2020).

### Research on Experiencing and Expressing Anger

Previous studies on mood disorder in patients with schizophrenia have mainly focused on either positive emotions or negative emotions such as sadness and fear. However, fewer studies have focused on anger. Anger is a negative emotion that people often experience in daily life. In order to quickly deal with threats and obstacles, anger modulates cognitive processes that detect danger quickly and respond to it (Izard, 2007). In contrast to other negative emotions that are characterized by low arousal and low pleasure, anger has high arousal and low pleasure (Russell and Barrett, 1999). Studying anger is important for schizophrenia because this disease is often associated with angry and hostile behavior (Volavka, 1999). Externalized anger in patients can lead to an increase in violent behavior (Brennan et al., 2000) and influence the prognostic process (Fassino et al., 2009). Therefore, this study focuses on anger experience and expression in patients with schizophrenia.

### Using fNIRS to Monitor Brain Activity in Emotion Experience and Expression

Emotions, including anger, are closely associated with the frontal cortex of the brain. The prefrontal cortex (PFC) is an important region for understanding and interpreting stimuli and their emotional meaning. Emotional stimuli activate the amygdala, but how emotions are expressed depends on the PFC. Anger can narrow the cognitive range and inhibit irrelevant information, impulses, and other emotional characteristics. Impulsiveness is based on damage to the PFC, and people with such damage show deficits in inhibiting emotion expression. Emotional euphoria and non-emotional responses are both associated with damage to the PFC (Meng, 2005). Studies have shown that the PFC is involved in emotion expression and feeling, and that patients with PFC impairment have significantly reduced emotion expression ability (Stuss et al., 1992). As such, in this study, we used functional near-infrared spectroscopy (fNIRS) to monitor the PFC activity of participants in the schizophrenia and healthy groups during emotion experience and expression.

fNIRS has been widely used in the evaluation of patients with schizophrenia in recent years (Balconi et al., 2015; Kumar et al., 2017; Chou et al., 2020). Compared with functional magnetic resonance imaging (fMRI) and electroencephalography, fNIRS has higher head movement tolerance, moderate time resolution and moderate spatial resolution, and a high ecological effect suitable for experiments carried out in more natural conditions (Ferrari and Quaresima, 2012; Pinti et al., 2020). In this study, given the uniqueness of the patient cohort consisting of individuals with schizophrenia, there were more random actions during the experiment, and the movement range of the participants in the drawing process was relatively large. Thus, fNIRS monitoring was more appropriate for this study as it could minimize the interference effect of these influencing factors.

Functional imaging has previously been used to explore the brain activity of study participants while they draw. Kaimal et al. (2017) used fNIRS to study the drawing process of study participants and found that the medial prefrontal cortex (mPFC) was activated during the drawing process compared to the resting state. The study also found that the activation of mPFC was strongest during doodling. Nakano et al. (2018) used fNIRS to assess and compare the activation of the PFC in patients with schizophrenia to that of healthy participants during the tree drawing test. The results showed that the blood oxygen level of the schizophrenia patients was significantly lower than that of the healthy participants during the tree drawing task. This study confirmed that differences in the tree drawing task may be related to brain dysfunction. Yan et al. (2021) used fNIRS to explore the role of drawing in regulating anger and sadness. The results showed that when drawing regulated sadness, the frontopolar area and left dorsolateral prefrontal cortex showed significant deactivations. These studies showed that fNIRS is an effective instrument to detect relative brain function.

### Research Questions and Hypothesis

In previous studies on anger, the following ways of priming emotions have been used: participants were asked to recall an experience that made them very angry (Small and Lerner, 2008); text, audio, video, and other external stimuli were presented to the participants (Carver, 2004; Barazzone and Davey, 2009); and emotional situational elicitation (Zheng et al., 2012) was used, where participants were asked to complete certain tasks and then given negative feedback to arouse anger (van’t Wout et al., 2010).

This study sought to assess the differences in ways and brain mechanisms of anger experience and expression through drawing between patients with schizophrenia and the healthy participants. Thus, a film was used as the activation material and a drawing task was used as the emotion expression tool. fNIRS was used to record brain activation so as to explore the similarities and differences between participants in the schizophrenia and healthy groups during the experience of anger and while using drawing to express emotions. In this study, we used a film clip to initiate anger, rather than recalling an angry experience (Small and Lerner, 2008), or emotional situational elicitation (van’t Wout et al., 2010), based primarily on the following considerations: the priming material should be sufficient and have group generality such as to allow the participants to fully experience anger and express the emotions they have experienced through drawing. The viewing time of the film clip is longer than that of pictures, and is episodic and therefore easier for people with schizophrenia to understand. Moreover, films combining both visual and auditory modalities are multi-modality eliciting materials, which are associated with better starting effects than single-modality eliciting materials (Gross and Levenson, 1995; Zheng et al., 2012). The main hypotheses of this study were associated with emotion experience and emotion expression, and were grouped as follows: (1) There are no significant differences in the experience of anger between participants in the schizophrenia and healthy groups; (1a) after experiencing anger, there are no significant differences between the two groups in the subjective ratings of emotion arousal and pleasantness; (1b) during the experience of anger, the activation levels of the PFC are significantly different between the two groups; (2) there are significant differences in the expression of anger between participants in the two groups, possibly because patients with schizophrenia have problems in expressing anger due to frontal lobe damage, express less emotion, and may exhibit weakened brain activation while drawing to express anger; (2a) there are significant differences in brain activation data between the two groups during the drawing process; (2b) there are significant differences between the two groups in terms of drawing features (e.g., line quality) and drawing content (e.g., the richness of the drawing); (2c) the ability of patients with schizophrenia to interpret the content of the drawing in words—i.e., their ability to relate the emotion expression of the drawing with the social meaning conveyed—is significantly different from that of healthy participants; (3) since drawing is a way for the participants to express their emotions, and the brain activation data during the drawing process are also recorded, there are correlations between the behavioral data and the brain activation data; (4) the drawing data and brain activation data during emotion expression are significantly correlated with each other, but the correlation patterns are different between participants in the schizophrenia and healthy groups.

According to the affective development model proposed by Halberstadt et al. (2001), emotions can be divided into different aspects—the ability to send affective messages, receive affective messages, and experience affect—that influence each other. There is a certain relationship between emotion experience and emotion expression. All of these factors can be used to derive Hypothesis (5): the drawing coding results and the arousal and valence being experienced are correlated significantly within each group.

## Materials and Methods

### Participants

This study has been approved by the University Committee on Human Research Protection of East China Normal University and the Ethics Committee of Shanghai Changning District Mental Health Center. The chief investigator informed the resident doctors in the mental health center of the screening conditions before the experiment, and the residents were asked to select those patients with schizophrenia who had been in the hospital for a long time. The residents determined the final list of patients who were to be included by communicating with the family members of the selected patients and obtaining their informed consent. Patients with schizophrenia in the convalescent period—i.e., hospitalized patients undergoing treatment who had their condition under control and were in a stable period after evaluation by psychiatrists—were enrolled using the following screening criteria: (1) patients who were right-handed, in good physical condition, and native Chinese speakers; (2) patients who had self-control and self-knowledge and could give independent informed consent; and (3) patients who possessed basic cognitive and communication functions and were able to understand instructions and cooperate with the completion of experimental tasks in the experiment. Patients were excluded if they met any of the following criteria: other serious physical illnesses such as diseases of the heart, liver, or kidney; intellectual disability or brain organic diseases; severe recession; impulsive and uncooperative; pregnant or lactating; and majored in art. A total of 25 participants were recruited in the study, but 8 were excluded due to data non-compliance (see section “fNIRS Data Analysis” for specific requirements). A total of 17 participants, including 8 males and 9 females, were finally included in the analysis (Table 1, see section “Positive and Negative Syndrome Scale” for details about PNASS scale and three subscales).

**TABLE 1:** Basic features of the two participant groups.

	Schizophrenia group	Healthy group	Significance
Number	17	18	
Age	37.56	35.11	NS
Gender (male/female)	8/9	6/12	
Education (years)	12.36	12.94	NS
PANSS	67.94 ± 9.65		
PANSS (Positive scale)	14.5 ± 4.147		
PANSS (Negative scale)	21 ± 3.521		
PANSS (General psychopathology scale)	32.38 ± 3.324		

*NS: not significant.*

The control group was recruited through advertisements. Inclusion criteria were as follows: (1) healthy participants with no history of mental illness, cardiovascular disease, brain injury, or substance abuse; (2) right-handed, in good physical condition, native Chinese speaker; (3) exclude participants majoring in art. A total of 24 control participants were recruited in this study, and 6 were excluded due to data non-compliance (see section “fNIRS Data Analysis” for specific requirements). A total of 18 participants were finally included in the analysis, including 6 males and 12 females (Table 1).

### Materials

#### Anger-Eliciting Material

The film clip The Tokyo Trial, with a runtime of 3 min and 51 s, was used as the anger-inducing material (see Supplementary Material Video for the film clip). The plot revolves around the Class-A war criminal, Hideki Tojo, who says during a trial that the massacre of the Chinese people was self-deserved. Several previous studies have reported that the experience of anger increased significantly in participants after watching the film clip (Ding et al., 2014; Shi et al., 2015; Shi, 2017).

#### The Affect Grid Scale

This scale is a self-reported single-item scale developed by Russell et al. (1989). Due to its simple and rapid scoring, it is mainly used for rapid and repeatable measurement of transient emotion states. The scale evaluates two dimensions of emotion: pleasure (valency) and arousal (arousal–sleepiness). Anger has high arousal and low pleasure on the scale.

This scale is a simple measurement method with good reliability and validity (Russell et al., 1989; Feldman Barrett and Russell, 1998), and has been used in many studies (Dalebroux et al., 2008; Diliberto-Macaluso and Stubblefield, 2015). However, due to the complexity of using this scale, a study prepared a video tutorial to teach participants how to use the scale (Askim and Knardahl, 2021). Another study has shown that the original single-response scale layout has no advantages compared to the scoring scale (Jaeger et al., 2021). In this study, in order to improve patient comprehension, the grid form was changed into a nine-point scale, and pleasure and arousal were split into two independent nine-point scores, providing multiple scoring options for each question (1: Displeasure, 9: Pleasure; 1: Sleepiness, 9: Arousal). The participants did not need to consider the binding relationship between the two.

#### Drawing Materials

The drawing materials consisted of an A4-sized white paper and oil drawing sticks in 24 colors. These materials were selected based on the relationship between materials and emotion expression in the ETC model (Hinz, 2018). According to this model, the properties of media are related to two continuums: emotion–cognitive experience and fluid–resistant medium. The oil drawing stick is closer to the cognitive end, and more appropriate for expressing emotions when participants are not completely immersed in emotions. Considering the hand-eye coordination and fine motor operation in schizophrenia and the age of the participants (mid-30s), the oil drawing stick was considered easier to handle and control than colored pencils and other fluid media. The text instructions were as follows: “Please express the emotions you just experienced when you watched the film. You can use any color you want and draw any pattern you want, as long as it reflects your emotions. The drawing time is 5 min, we will remind you when the time is up, please try not to stop during the drawing.”

#### Drawing Open-Ended Self-Reflection Questionnaire

A self-report questionnaire was provided, containing five open-ended questions about the following aspects of the drawing: “What are your emotions when you draw? Use 3–5 adjectives to describe it,” “What did you draw?” “What do you think of when you look at the picture after you finish drawing it?” “Is there any connection between the state presented in the picture and your reality?” and “Give the picture a name.” The fourth question tested whether the participants could relate the emotional expression of the drawing with the social meaning it conveyed.

#### Positive and Negative Syndrome Scale

The positive and negative syndrome scale (PANSS) (Kay et al., 1987) is a 30-item scale that assesses the symptoms of schizophrenia and each item is rated on a 7-point scale (1: absent, 7: extreme). It includes three subscales: the positive scale (7 items, total scores from 7 to 49), the negative scale (7 items, total scores from 7 to 49) and the general psychopathology scale (16 items, total scores from 16 to 112). Thus, the PANSS total score ranges from 30 to 210. Additionally, “mildly ill” corresponds to a PANSS total score of 58, “moderately ill” to a PANSS score of 75, “markedly ill” to a PANSS score of 95, and severely ill to a PANSS score of 116 (Leucht et al., 2005).

#### fNIRS Apparatus

**FIGURE 1:**
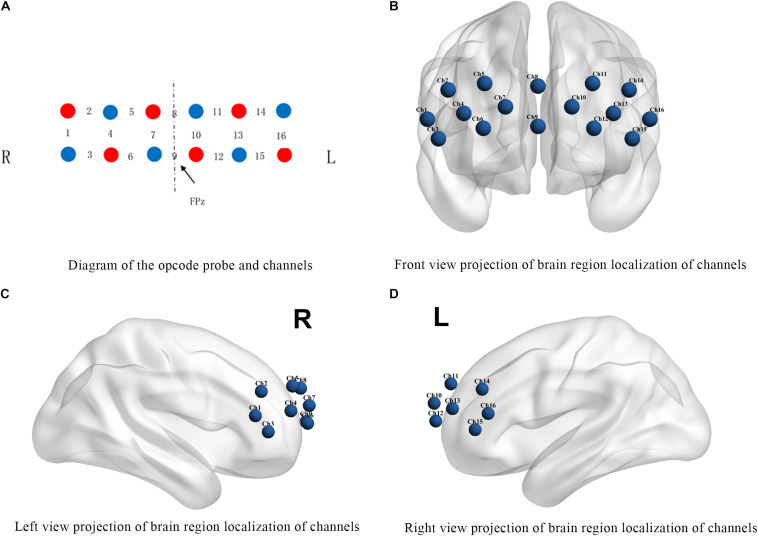
Channel positions. **(A)** The red circles represent the light transmitting probes and the blue circles represent the light receiving probes. The numbers between probes indicate the signal channel numbers. R: right brain region; L: left brain region. **(B–D)** Show the projections of brain region localization of channels.

A 16-channel portable fNIRS system WOT-100 (Hitachi Medical Co., Ltd., Japan) was used to collect cerebral blood flow data. The instrument emits and absorbs near-infrared light at two wavelengths, 705 nm and 830 nm, with a sampling rate of 5 Hz. The system consists of 6 light-transmitting probes and 6 receiving probes with a total of 16 channels covering the PFC (Figure 1), and the anatomical position of each channel is listed in Table 2.

**TABLE 2:** Broadman areas, brain regions, and the probability of corresponding fNIRS channels.

Broadman areas	Brain areas	Channels		Probability
BA44/45	Broca’s area	1	16	1
		2	14	0.818
		3	15	0.537
BA46/9	Dorsolateral prefrontal cortex (DLPFC)	4	13	0.741
		5	11	0.736
		3	15	0.463
		8		0.21
		2	14	0.182
BA10	Frontopolar area	7	10	1
		9		1
		6	12	0.881
		8		0.79
		5	11	0.264
		4	13	0.259
BA11	Orbitofrontal area	6	12	0.119

### Study Procedure

**FIGURE 2:**
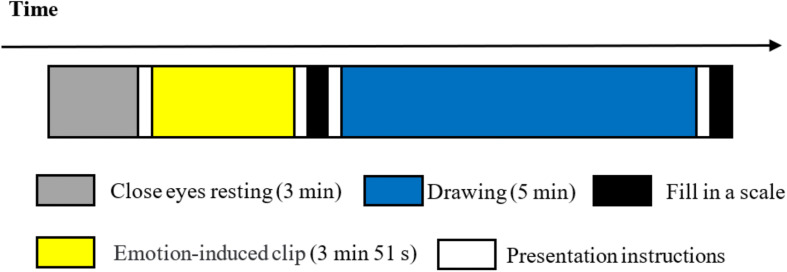
Experimental procedure.

One week prior to the experiment, resident doctors assessed the mental status of the experimental group using PANSS. When participants entered the laboratory at the beginning of the formal experiment, the principal experimenter introduced himself to the participants, explained the whole study, and obtained written informed consent from the participants. Next, the experimenter explained the specific content and process of the experiment and introduced the use of the art medium and emotion grid scale. After ensuring that the participants understood all the information, they were equipped with portable near-infrared devices. Following the successful debugging of all channels, the experiment was formally initiated. The experimental procedure (Figure 2) was as follows: first, the participants closed their eyes and rested for 3 min. Then they watched the anger-inducing film clip. After the film clip had ended, the participants filled out the paper version of the affect grid scale with a pen, and then drew with oil drawing sticks for 5 min, then answered the paper version of the open-ended self-reflection questionnaire with a pen. All the writing materials (the affect grid scale, paper for drawing, and the drawing introspection scale) were clamped onto an A4-sized board and presented in vertical orientation to the participants at the beginning of the experiment. The instructions were all timed by the computer.

### Triangulation in Research Design, Implementation, and Data Analysis

According to the triangulation method proposed by Denzin (2017), combining multiple methods, empirical viewpoints, experimental hypotheses, and researchers in a single study can increase the rigor, depth, and breadth of the study. Following the classification suggested by Denzin (2017), this study adopted a triangulation strategy in several aspects: theoretical triangulation using multiple professional perspectives to interpret a single set of data or information (e.g., both brain activation data and behavioral data were used to interpret the hypotheses); methodological triangulation, including inter-method triangulation by adopting quantitative and qualitative methods simultaneously, and intra-method triangulation by using two or more similar methods to measure the same variable (e.g., the analysis of drawing contents and answers to reflection question); data triangulation, in which data were collected from multiple sources and each hypothesis was tested by brain activation data and behavioral indicators; and investigator triangulation, i.e., joint research by more than one skilled investigator without prior discussion or cooperation. In this study, both brain and drawing data were analyzed by different researchers and the drawing data were coded independently by two researchers.

### Data Analysis

#### Data Analysis of Emotional Changes

The affect grid scale was used to measure the subjective emotional feelings of the participants at the end of the emotion-inducing film. A one-sample *t*-test with a test value of 5 (mid-point of scale) was used to analyze the changes in emotion—pleasure and arousal—among the two groups of participants. The two-sample *t*-test was used to compare the differences of pleasure and arousal between the two groups.

#### fNIRS Data Analysis

The fNIRS data were preprocessed using the NIRS_SPM kit (Ye et al., 2009) based on MATLAB. Oxygenated hemoglobin (HbO) is the most sensitive marker of changes in blood flow in the brain (Hoshi et al., 2001) with high signal-to-noise ratio (Zhang et al., 2018). Therefore, HbO concentration was selected for analysis in this study. Prior to preprocessing, signal-free and noise channels (correlation coefficient of HbO and HbR higher than 0.5) were eliminated. If the number of channels removed accounted for more than 25% of the total number of channels (i.e., 4 or more of the 16 channels in this study), the participant was removed from the analysis (McDonald et al., 2019). Correlation-based signal improvement (Cui et al., 2010) was used to correct artifacts caused by head movement. The wavelet-minimum description length and hemodynamic response functions methods were used to remove the signal drift caused by physiological and machine noises (Jang et al., 2009; Tak and Ye, 2014).

For each individual participant, the average HbO concentration change of 16 channels during the task period, including the 3-min closed-eye rest, 3-min-and-51-s emotion-inducing film task, and 5-min drawing task, was extracted. The 3-min closed-eye rest before the experiment was used as the baseline (Jiang et al., 2012; Shin et al., 2016), and the HbO concentration values during the tasks minus baseline value were used as indicators of brain activation patterns. A one-sample *t*-test with a test value of 0 was used to analyze the activation patterns of the PFC during the emotion-inducing film task and drawing task (Baker et al., 2016). Following this, a two-sample *t*-test was conducted to analyze the differences in brain activation patterns between the two groups of participants. To analyze the dynamic changes in differences between the two groups in the emotional induction stage, a two-sample *t*-test was also conducted on the degree of brain activation per second in the emotional induction stage. Data were visualized using the xjview^1^ software and the BrainNet Viewer toolbox^2^ (Xia et al., 2013). The false discovery rate (FDR) was used to correct the *p* values of multiple tests (Singh and Dan, 2006).

#### Drawing Coding Indicator Analysis

The coding process was a round-trip iteration. According to the research objectives, a researcher determined the drawing coding indicators: drawing layout, color characteristics, line and stroke characteristics, drawing content, and text description of the drawing. The first four coded the features and content of the drawing, and the last indicator represented the process of the participants interpreting the drawing. The researchers identified 17 codes based on these coding indicators. After defining the operation, two researchers independently coded all the drawings. The final coding manual is shown in Supplementary Table 1. Apart from the completely consistent coding indicator, paper orientation, Intraclass Correlation Coefficients (ICC) analysis was conducted to test the consistency between raters for the remaining 16 ordered polytomous coding indexes, and internal consistency analysis was performed for all coding of each rater. A high level of consistency was seen among the raters for all drawing coding indicators (0.766 ≤ α ≤ 1.000) in both groups (Table 3), as well as for the responses given by the individual subjects (α = 0.823, 0.845, respectively). Data were analyzed using the means of drawing coding indicators rated by two distinct raters.

**TABLE 3:** Consistency of ratings for drawing coding indicators.

	Healthy group (*n* = 17)	Schizophrenia group (*n* = 18)
	
	ICC	ICC
Paper orientation	1.000	1.000
Tinge	0.994**	0.990**
The number of colors	0.998**	0.997**
Line fluency	0.843**	0.985**
Line variability	0.910**	0.925**
Line quality	0.766*	0.958**
Pressure	0.889**	0.948**
Number of drawing elements	1.000	0.997**
Drawing space	0.990**	0.991**
Richness of the drawing	0.952**	0.971**
Intensity of the emotion expressed in drawings	0.941**	0.985**
Clarity of the theme of the drawing	0.953**	0.986**
Richness of literal description	1.000	0.994**
Meaningfulness of the literal description	0.963**	0.954**
Relation between wording and the participant’s reality	1.000	0.921**
Quality and symbolism of the title	0.953**	0.951**
Consistency between drawing and literal words	0.878**	0.961**

ICC: Interclass correlation coefficient; * p < 0.05, ** p < 0.01.

## Results

### Emotion Experience

#### Emotion Induced

The one-sample *t*-test on the affect grid scale scores of the two groups revealed that all the participants experienced a higher degree of arousal (*M* = 6.43, *p* < 0.01) and a lower degree of pleasure (*M* = 3.34, *p* < 0.01), indicating that the effect of the film clip on anger was significant. The affect grid scale scores of the two groups were further analyzed and compared. The two-sample *t*-test results showed that there was no significant difference between the two groups in the scores for arousal and pleasure (Table 4). Based on these results, Hypothesis 1a was verified.

**TABLE 4:** Emotions induced.

	Arousal		Pleasure	
	Mean	*t*	Mean	*t*
All participants	6.43	3.709**	3.34	−4.669**
Schizophrenia group	6.12	–0.774	3.76	1.16
Healthy group	6.72		2.94	

** p < 0.01.

#### Activation of Brain Regions During Emotion Experience

The normality test of brain activation data showed that most of the data did not follow normal distribution (for more details, see Supplementary Table 2); therefore, bootstrap sampling was conducted 5000 times (Preacher and Hayes, 2008) before analysis. A one-sample *t*-test was conducted for HbO concentrations of the two groups of participants while they were being shown the film (Supplementary Table 3). Participants in the experimental group had significantly negative activation in all recorded brain regions, except in a part of the left Broca’s area (Ch16). Participants in the control group had significantly negative activation in the Broca’s area, DLPFC and frontopolar area (Ch2, Ch4, Ch5, Ch7, Ch9, Ch10, Ch11, Ch12, Ch13, and Ch15). These results indicated that both groups reacted to the movie clips. A two-sample *t*-test was conducted to compare HbO concentrations of the two groups at this stage. There were no significant differences in any channels between the experimental group and the control group, indicating that patients with schizophrenia could experience anger normally. Thus, Hypothesis 1b was verified.

**FIGURE 3:**
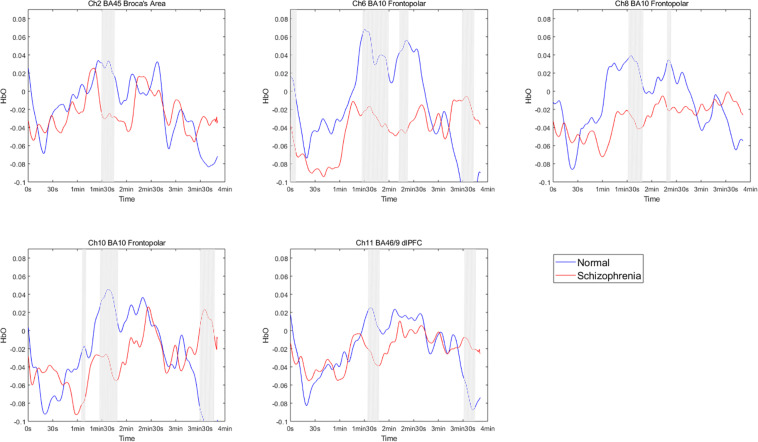
HbO concentration per second changes in participants while watching the emotion-inducing film. The segments with a gray line represent significant differences between the two groups.

The brain activation data of the two groups for the entire duration of watching the film were further compared. The results showed significant differences between the groups in some channels during this period, and significant differences in the frontopolar area, DLPFC and right Broca’s area (Ch2, Ch6, Ch8, Ch10, and Ch11) at partial time points (Figure 3). This result supplements Hypothesis 1b. Although there were no significant differences in average brain activation between the two groups for the entire duration of the emotion experience stage, there were significant differences during some periods.

### Emotion Expression

#### Activation of Brain Regions During Emotion Expression

**FIGURE 4:**
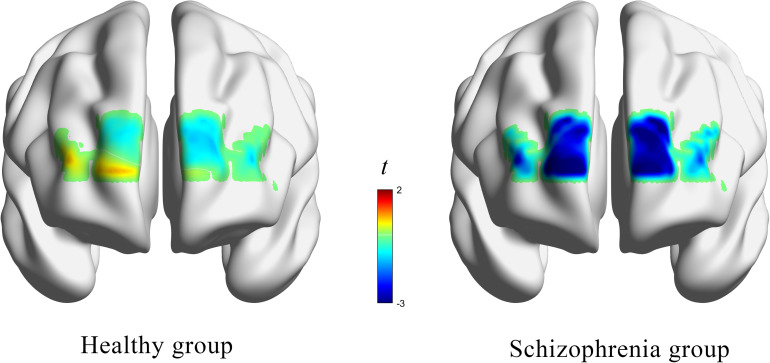
The *t* value (compared to 0) of brain activation in participants during emotion expression through drawing.

**FIGURE 5:**
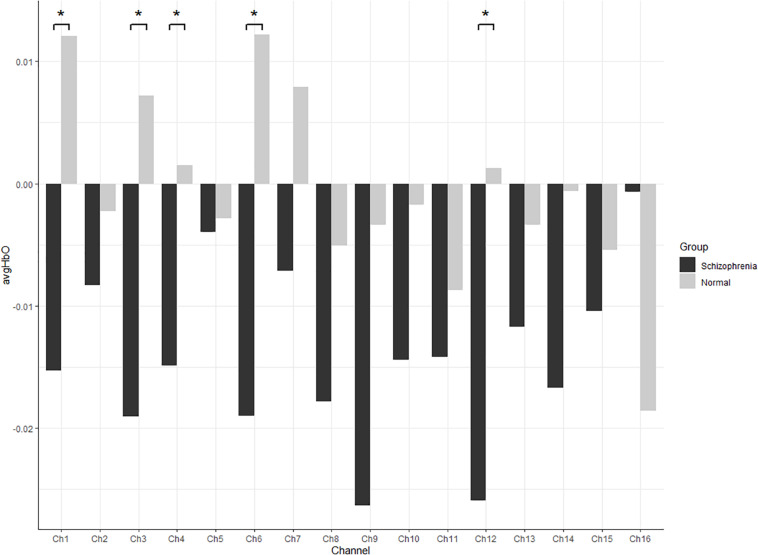
All channels HbO concentrations in participants during emotion expression through drawing (* refer significant difference between two groups *p* < 0.05).

HbO concentrations during the stage of emotion expression were analyzed for participants in the two study groups (Figure 4 and Supplementary Table 4). Participants in the experimental group had significant negative activation in the Broca’s area, DLPFC, and frontopolar and orbitofrontal areas (Ch1, Ch3, Ch4, Ch6, Ch8, Ch9, Ch10, Ch11, Ch12, and Ch14), whereas those in the control group had no significant activation area. A two-sample *t*-test revealed significant differences between the groups in the frontopolar, orbitofrontal, and right Broca’s areas and in the right DLPFC (Ch1, Ch3, Ch4, Ch6, and Ch12). Based on these results (Table 5 and Figure 5), Hypothesis 2a was verified.

**TABLE 5:** Two-sample *t*-test results for the comparison of brain activation patterns in each channel during emotional expression between the two participant groups.

Channel	*t*	*p (fdr corrected)*
Ch1	–3.055	0.026*
Ch2	–0.991	0.439
Ch3	–3.091	0.026*
Ch4	–2.660	0.038*
Ch5	–0.172	0.864
Ch6	–2.906	0.026*
Ch7	–1.482	0.215
Ch8	–1.967	0.115
Ch9	–2.355	0.067
Ch10	–1.546	0.211
Ch11	–0.784	0.501
Ch12	–2.929	0.026*
Ch13	–0.882	0.473
Ch14	–1.830	0.136
Ch15	–0.566	0.614
Ch16	1.999	0.115

df = 33,* p < 0.05, ** p < 0.01.

#### Emotion Expression Through Drawing

##### Group differences in drawing coding indicators

**FIGURE 6:**
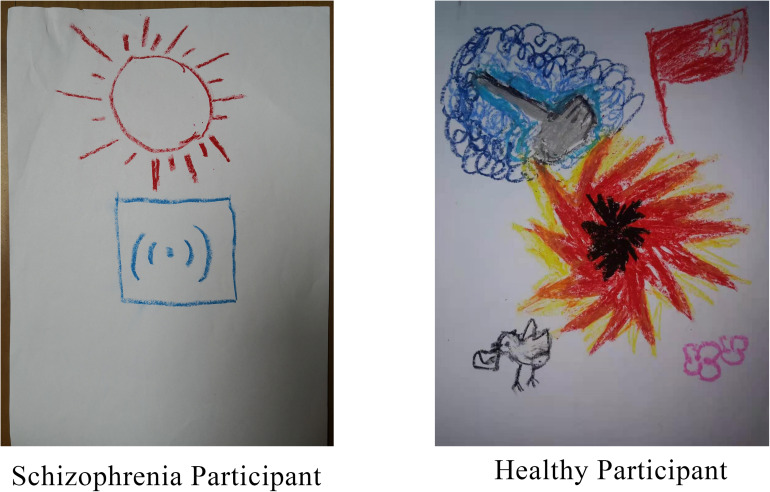
Specific examples of drawings from participants in the schizophrenia group (left) and the healthy group (right).

To assess differences in the coding of drawings between the two groups, the chi-squared test was carried out using group as an independent variable and paper direction as a dependent variable. More than two-thirds of the participants drew vertically, and there was no significant group difference in the orientation of the paper. This may have been because the paper was handed to the participants in the vertical orientation. The normality test of other picture coding data showed that most of the data did not follow normal distribution (for more details, see Supplementary Material); therefore, bootstrap sampling was conducted 5000 times (Preacher and Hayes, 2008) before analysis. We further used different groups as independent variables and drawing coding indicators as dependent variables for a *t*-test. Results demonstrated that except for the tinge, color number, stroke weight, and a marginal significance of drawing size, there were significant differences in other drawing features. The line change, number of elements in the drawing, drawing content richness, emotional intensity expressed by the drawing, and the clarity of drawing theme were significantly different between the two groups. Hypothesis 2b was thus partially confirmed. In addition to the marginal significance of the richness of the text description, there were significant group differences in the text description, the connection between the text description and the artist’s reality, the quality and symbolism of the title, and consistency between the drawing and text. Hypothesis 2c was partially verified (Table 6). Figure 6 shows specific examples of drawings from participants in the two groups.

**TABLE 6:** Comparison of drawing coding results among different groups.

Indicators	All participants (*n* = 35)	Healthy group (*n* = 18)	Schizophrenia group (*n* = 17)	Test statistic	*p*	Effect size (*d*) [95% CI]
Paper orientation				*χ^2^* = 1.47	0.238	0.41[−0.27,1.08]
1 = Horizontal	7 (17.10%)	5 (27.80%)	2 (11.80%)			
2 = Vertical	28 (68.30%)	13 (72.20%)	15 (88.20%)			
Tinge	3.46 ± 1.40	3.08 ± 1.48	3.85 ± 1.22	*t* = 1.67	0.104	0.56[−0.11,1.24]
Number of colors	3.67 ± 2.37	3.97 ± 2.37	3.35 ± 2.40	*t* = 0.77	0.447	0.26[−0.41, 0.93]
Line fluency	4.00 ± 1.28	4.67 ± 0.59	3.29 ± 1.44	*t* = 3.73	0.001**	1.26[0.54,1.99]
Line variability	3.59 ± 1.27	4.31 ± 0.86	2.82 ± 1.21	*t* = 4.19	0.000**	1.42[0.68,2.16]
Line quality	3.46 ± 1.17	4.06 ± 0.78	2.82 ± 1.20	*t* = 3.62	0.001**	1.22[0.50,1.95]
Pressure	3.73 ± 0.74	3.83 ± 0.49	3.62 ± 0.94	*t* = 0.86	0.397	0.29[−0.38,0.96]
Number of drawing elements	4.93 ± 3.17	5.94 ± 3.59	3.85 ± 2.29	*t* = 2.04	0.049*	0.69[0.01,1.37]
Drawing space	0.74 ± 0.27	0.82 ± 0.19	0.65 ± 0.31	*t* = 1.98	0.056	0.67[0.01,1.35]
Richness of the drawing	3.09 ± 1.36	3.69 ± 3.69	2.44 ± 1.26	*t* = 3.04	0.005*	1.03[0.32,1.73]
Intensity of the emotion expressed in drawings	2.86 ± 1.38	3.75 ± 1.06	1.91 ± 1.00	*t* = 5.26	0.000**	1.78[1.00,2.56]
Clarity of the theme of the drawing	2.89 ± 1.53	3.92 ± 1.19	1.79 ± 1.02	*t* = 5.65	0.000**	1.91[1.11,2.71]
Richness of literal description	3.50 ± 1.59	4.00 ± 1.41	2.97 ± 1.62	*t* = 2.00	0.053	0.68[-0.01,1.36]
Meaningfulness of the literal description	3.27 ± 1.46	4.19 ± 1.09	2.29 ± 1.13	*t* = 5.07	0.000**	1.71[0.94,2.49]
Relation between wording and the participant’s reality	3.06 ± 1.61	4.17 ± 1.20	1.88 ± 1.07	*t* = 5.93	0.000**	2.01[1.19,2.81]
Quality and symbolism of the title	3.20 ± 1.48	4.22 ± 1.09	2.12 ± 1.00	*t* = 5.97	0.000**	2.02[1.20,2.83]
Consistency between drawing and literal words	3.39 ± 1.59	4.72 ± 0.49	1.97 ± 1.00	*t* = 10.49	0.000**	3.55[2.48,4.61]

Numbers indicate sample size and percentage (n(%)) or mean ± standard deviation (M ± SD). CI: confidence interval,* p < 0.05, ** p < 0.01.

##### Correlation analysis of brain data between drawing coding and emotion expression

Scatter plots were prepared using the drawing coding data as the abscissa coordinate and the brain data of the two groups as the vertical coordinate. The fitted results showed that the drawing coding indicators were linearly correlated with brain data in the two groups. Since most of the brain data and picture coding data do not conform to the normal distribution, spearman correlation analysis was carried out between drawing coding and brain activation data in healthy participants during the stage of emotion expression. Results revealed that in the drawing stage, the frontopolar area, DLPFC, and right Broca’s area (Ch2 and Ch8) were correlated with more than five drawing coding indicators in the control group. Specifically, both channels were significantly positively correlated with line variability, richness of literal description, and meaningfulness of the literal description. Paper orientation, tinge, number of colors, line fluency, pressure, relation between wording and the painter’s reality, and consistency between drawing and literal words were significantly correlated to only zero or to one channel. The other codes were all significantly correlated to brain data in two or more channels, of which meaningfulness of the literal description was significantly correlated with five channels (Table 7).

**TABLE 7:** Correlation coefficients between drawing coding and brain data in the healthy group during the emotional expression stage.

	Δ	Δ	Δ	Δ	Δ	Δ	Δ	Δ	Δ	Δ	Δ	Δ	Δ	Δ	Δ	Δ
	draw_ch1	draw_ch2	draw_ch3	draw_ch4	draw_ch5	draw_ch6	draw_ch7	draw_ch8	draw_ch9	draw_ch10	draw_ch11	draw_ch12	draw_ch13	draw_ch14	draw_ch15	draw_ch16
Tinge	0.129	0.400	0.232	0.140	–0.003	0.164	0.109	0.180	–0.033	–0.082	0.014	0.042	0.151	–0.004	0.072	0.513*
The number of colors	0.288	0.529*	–0.101	0.053	0.187	0.030	–0.005	0.385	–0.249	0.279	0.311	0.006	0.075	0.278	–0.115	0.467
Line fluency	0.262	0.143	0.104	0.219	–0.052	0.233	–0.023	0.349	–0.015	0.137	0.452	–0.036	0.382	0.062	0.467	0.166
Line variability	0.340	0.610**	–0.091	0.237	0.043	0.129	0.016	0.609**	0.150	0.345	0.474*	–0.158	0.249	0.093	0.080	0.293
Line quality	0.002	0.213	–0.172	–0.010	0.112	0.131	–0.172	0.455	0.100	0.354	0.605**	–0.129	0.117	0.311	–0.100	0.085
Pressure	–0.198	–0.215	–0.189	–0.349	–0.384	–0.145	–0.419	−0.507*	–0.360	–0.354	–0.134	–0.137	–0.348	0.114	–0.211	0.023
Number of drawing elements	0.472*	0.707**	0.210	0.253	0.048	0.037	0.196	0.383	–0.154	0.245	0.107	0.126	0.353	0.093	0.170	0.161
Drawing space	0.115	0.357	0.324	0.229	0.509*	0.157	0.352	0.526*	–0.024	0.590**	0.545*	0.359	0.275	0.339	0.367	–0.072
Richness of the drawing	0.526*	0.603**	0.090	0.106	–0.072	–0.050	0.034	0.465	–0.164	0.265	0.338	–0.025	0.285	0.100	0.184	0.219
Intensity of the emotion expressed in drawings	0.530*	0.345	0.428	0.183	0.026	0.352	0.263	0.336	0.048	0.316	0.283	0.291	0.390	0.215	0.645**	0.262
Clarity of the theme of the drawing	0.304	0.522*	0.221	0.298	0.179	0.449	0.219	0.449	0.260	0.417	0.446	0.342	0.584*	0.409	0.386	0.176
Richness of literal description	0.432	0.622**	0.121	0.434	0.359	0.351	0.297	0.596**	0.530*	0.526*	0.193	0.158	0.338	0.196	0.196	0.393
Meaningfulness of the literal description	0.398	0.493*	0.250	0.442	0.196	0.582*	0.277	0.520*	0.506*	0.511*	0.241	0.240	0.464	0.356	0.333	0.281
Relation between wording and the participant’s reality	0.039	0.197	0.084	0.009	0.446	0.325	0.139	0.248	0.593**	0.348	0.125	0.343	0.051	0.308	0.161	0.409
Quality and symbolism of the title	0.115	0.430	0.138	0.157	0.096	0.212	0.160	0.494*	0.580*	0.296	0.179	–0.035	0.179	–0.041	0.085	0.122
Consistency between drawing and literal words	0.015	0.318	0.279	0.217	0.064	0.448	0.030	0.332	0.234	0.225	0.130	0.083	0.368	0.148	0.221	–0.013

“Δ draw” indicates brain activation in each channel during the drawing task. * p < 0.05, ** p < 0.01.

The same analysis was also carried out for data obtained from patients with schizophrenia (for detailed normal distribution test results refer to Supplementary Table 2). The results showed that the frontopolar area, DLPFC, left Broca’s area, and left orbitofrontal area (Ch8, Ch9, Ch10, Ch12, and Ch15) were associated with more than five (including five) drawing coding indexes during the drawing stage. Paper orientation, tinge, number of colors, meaningfulness of the literal description, quality and symbolism of the title, and consistency between drawing and literal words were significantly correlated with the data of only one or zero brain channels. Line quality, drawing space, intensity of the emotion expressed in the drawings, and clarity of the theme of the drawing were significantly correlated with more than five channels (Table 8). Hypothesis 3 was thus partially verified.

**TABLE 8:** Correlation coefficients between drawing coding and brain data in the schizophrenia group during the emotional expression stage.

	Δ	Δ	Δ	Δ	Δ	Δ	Δ	Δ	Δ	Δ	Δ	Δ	Δ	Δ	Δ	Δ
	draw_ch1	draw_ch2	draw_ch3	draw_ch4	draw_ch5	draw_ch6	draw_ch7	draw_ch8	draw_ch9	draw_ch10	draw_ch11	draw_ch12	draw_ch13	draw_ch14	draw_ch15	draw_ch16
Tinge	–0.396	0.200	0.291	0.040	0.126	0.317	0.176	0.246	0.260	0.342	0.445	0.540*	0.233	0.026	0.481	–0.153
The number of colors	0.182	–0.260	–0.040	–0.131	–0.317	–0.338	–0.230	–0.360	–0.248	–0.236	–0.151	–0.316	–0.198	–0.234	–0.477	–0.053
Line fluency	–0.032	–0.041	−0.582*	–0.048	–0.170	–0.237	–0.297	–0.170	−0.566*	–0.371	–0.276	–0.459	−0.495*	–0.156	−0.693**	–0.393
Line variability	–0.142	–0.259	–0.417	–0.265	–0.248	–0.445	–0.161	–0.130	−0.492*	–0.439	–0.345	−0.546*	−0.617**	–0.345	−0.655**	–0.452
Line quality	–0.120	–0.176	–0.432	–0.216	–0.297	–0.240	–0.369	–0.324	−0.588*	−0.490*	–0.392	−0.532*	−0.559*	–0.209	−0.719**	–0.324
Pressure	0.022	–0.264	–0.334	–0.356	–0.295	−0.508*	–0.387	−0.526*	–0.221	–0.453	–0.338	–0.383	–0.302	–0.395	–0.036	–0.025
Number of drawing elements	0.330	−0.573*	–0.021	–0.159	−0.511*	–0.272	–0.355	–0.470	–0.440	–0.398	–0.440	–0.233	–0.314	–0.245	−0.602*	0.025
Drawing space	–0.033	–0.414	–0.427	–0.045	−0.589*	–0.380	−0.497*	−0.567*	−0.577*	−0.687**	−0.719**	−0.642**	−0.677**	–0.468	−0.636**	–0.227
Richness of the drawing	0.184	–0.256	–0.322	0.008	–0.376	–0.403	–0.398	–0.446	−0.568*	–0.453	–0.466	−0.547*	–0.468	–0.288	−0.808**	–0.111
Intensity of the emotion expressed in drawings	0.097	−0.607**	–0.218	–0.217	–0.426	–0.270	−0.606**	−0.490*	−0.600*	−0.652**	–0.469	–0.345	–0.272	–0.264	–0.426	0.008
Clarity of the theme of the drawing	–0.012	–0.452	–0.190	–0.143	−0.502*	–0.461	−0.645**	−0.532*	−0.484*	−0.552*	–0.320	–0.229	–0.324	–0.349	–0.423	–0.153
Richness of literal description	0.097	–0.438	–0.388	–0.456	–0.446	–0.449	–0.460	−0.631**	−0.528*	−0.505*	–0.243	–0.136	–0.106	–0.290	–0.374	–0.125
Meaningfulness of the literal description	0.008	0.011	–0.245	–0.152	–0.095	–0.096	–0.302	–0.342	–0.429	–0.168	0.063	0.069	0.159	–0.188	–0.058	–0.107
Relation between wording and the participant’s reality	0.360	–0.391	–0.150	–0.279	–0.353	–0.431	–0.223	−0.502*	−0.685**	–0.225	–0.324	–0.254	0.054	–0.363	–0.302	0.014
Quality and symbolism of the title	0.285	0.029	–0.347	–0.023	0.014	–0.086	–0.183	–0.308	–0.404	–0.060	0.080	0.016	0.225	–0.168	–0.109	0.055
Consistency between drawing and literal words	–0.021	–0.167	0.118	–0.283	–0.072	–0.276	–0.146	–0.239	–0.243	–0.057	0.192	0.215	0.274	–0.177	0.028	–0.006

“Δ draw” indicates brain activation in each channel during the drawing task. * p < 0.05, ** p < 0.01.

##### Correlation analysis of drawing coding with participants’ background information and self-rating scores

Scatter plots were prepared using the drawing coding data as the abscissa coordinate and the self-rated arousal and valence scores of the two groups (for patients with schizophrenia, extra disease information was included) as the vertical coordinate. The fitted results showed that the drawing coding indicators were linearly correlated with self-rated arousal and valence scores of the two groups (and the disease information of schizophrenia patients). And normality test of data showed that most of them did not conform to the normal distribution. Therefore, linear spearman correlation analysis was conducted between drawing coding indicators and self-rated arousal and pleasure scores of the control group after they had watched the film. The results showed a significant positive correlation between arousal and tinge (*r* = 0.499, *p* = 0.035) and a significant negative correlation between arousal and drawing space (*r* = −0.606, *p* = 0.008). In other words, the higher the emotional arousal of healthy participants, the warmer the tinge and the smaller the drawing space used on the paper. There were significant correlations between pleasure and intensity of the emotion expressed in drawings (*r* = −0.683, *p* = 0.002). Specifically, the lower the valence of the participants after watching the film, the higher the intensity of the emotion expressed in drawings.

For patients with schizophrenia, linear correlation analysis was conducted between drawing coding indicators and PANSS (negative) scores, PANSS (positive) scores, general psychopathology, course of disease, and self-rated arousal and pleasure. The results showed no significant correlations between arousal and coding indexes. There was a significant positive correlation between pleasure and line quality (*r* = 0.486, *p* = 0.048), indicating that the happier the patients were after watching the film, the higher the quality of the drawing lines was. PANSS (negative) scores were positively correlated with the number of colors (*r* = 0.532, *p* = 0.034) while PANSS (positive) scores were significantly correlated with tinge (*r* = −0.535, *p* = 0.033), number of colors (*r* = 0.602, *p* = 0.014), and number of drawing elements (*r* = 0.512, *p* = 0.043), indicating that schizophrenia patients with more symptoms regardless of valence used more kinds of colors but when patients were with more positive symptoms, they used more and colder colors and more elements for drawing. The course of the disease was significantly negatively correlated with the richness of literal description (*r* = −0.603, *p* = 0.022) and relation between wording and the participant’s reality (*r* = −0.557, *p* = 0.039); thus, a longer illness indicated less verbal description of the drawing and a weaker relation between wording and the participant’s reality (Table 9). From these results, Hypothesis 4 was partially verified.

**TABLE 9:** Linear correlation coefficients between drawing coding indicators, self-rated arousal and pleasure scores, and disease information.

	Healthy group (*n* = 18)		Schizophrenia group (*n* = 17)					
	Arousal	Pleasure	Arousal	Pleasure	PANSS (negative)	PANSS (positive)	General psychopathology	Course of disease
Paper orientation	–0.183	–0.224	–0.209	–0.303	–0.351	–0.433	–0.474	0.025
Tinge	0.499*	0.003	0.074	–0.055	–0.073	−0.535*	–0.184	–0.074
Number of colors	0.212	–0.134	–0.307	0.342	0.532*	0.602*	0.642**	0.111
Line fluency	–0.108	–0.397	0.027	0.300	–0.164	0.061	0.015	0.007
Line variability	–0.152	–0.100	0.236	0.331	–0.031	0.310	0.188	0.016
Line quality	–0.417	0.104	0.023	0.486*	0.077	0.319	0.313	–0.012
Pressure	0.213	0.038	0.377	0.289	–0.207	–0.091	–0.183	–0.198
Number of drawing elements	0.361	–0.303	–0.217	0.294	0.227	0.512*	0.482	–0.173
Drawing space	−0.606**	–0.384	0.177	0.043	–0.284	0.243	0.049	–0.426
Richness of the drawing	0.146	–0.283	–0.155	0.294	0.144	0.473	0.418	–0.194
Intensity of the emotion expressed in drawings	0.212	−0.683**	0.482	–0.229	–0.387	0.102	–0.118	–0.470
Clarity of the theme of the drawing	0.130	–0.349	0.459	–0.026	–0.365	–0.209	–0.042	–0.527
Richness of literal description	0.129	–0.154	0.338	–0.046	–0.320	–0.219	–0.138	−0.603*
Meaningfulness of the literal description	0.160	–0.303	0.309	0.224	–0.305	–0.367	–0.022	–0.394
Relation between wording and the participant’s reality	0.112	–0.097	0.256	0.247	–0.158	0.209	0.035	–0.369
Quality and symbolism of the title	–0.138	0.098	0.055	–0.001	–0.280	–0.205	–0.176	–0.399
Consistency between drawing and literal words	–0.052	0.050	0.308	0.173	0.029	–0.126	0.158	–0.327

PANSS: positive and negative syndrome scale, * p < 0.05, ** p < 0.01.

## Discussion

In this study, a movie clip was used to elicit emotions while drawings were used as a way for participants to express emotions. Through the scale and brain activation result data, we verified that there was no significant difference between schizophrenia patients and healthy participants in the experience of anger elicited by the film clip. However, in the expression of anger through drawing, brain activation and drawing results were found to differ significantly between the schizophrenia and healthy groups. The differences in anger expression were evident in the brain data, drawing contents, and interpretation of the drawings. Patients with schizophrenia showed significant negative activation in the Broca’s area, DLPFC, and frontopolar and orbitofrontal areas (Ch1, Ch3, Ch4, Ch6, Ch8, Ch9, Ch10, Ch11, Ch12, and Ch14) whereas those in the control group had significant negative activation in a part of the left Broca’s area (Ch16). We also found significant group differences in the drawing content including in the line variability, the weight of strokes, the number of drawing elements, the richness of the drawing, the intensity of the emotion expressed in drawings, and the clarity of the theme of the drawing. Significant group differences in the wording to describe the drawing were evident in the richness of the literal description, its meaningfulness, the relation between wording and the participant’s reality, the quality and symbolism of the title, and the consistency between the drawing and literal words. In addition, there were significant correlations between emotion experience and emotion expression in the two groups. Correlation analysis of drawing coding and brain activation data showed that the DLPFC and right Broca’s area (Ch2 and Ch8) in the healthy group and the frontopolar area, DLPFC, left Broca’s area, and left orbitofrontal area (Ch8, Ch9, Ch10, Ch12, and Ch15) in patients with schizophrenia were correlated with multiple drawing coding indexes. The results also demonstrated that the emotional intensity of drawings was significantly correlated with the subjective evaluation index of emotion.

### Experience of Anger

The results of the affect grid scale showed that both patients with schizophrenia and the healthy group experienced higher arousal and lower pleasure without significant differences in the scores of the two groups. This suggests that both patients with schizophrenia and healthy people experienced anger through watching the movie clips without differences in the degree of anger. This result is similar to that of a previous meta-analysis (Berenbaum and Oltmanns, 1992; Reske et al., 2007; Yan et al., 2012). Changes in HbO concentration during the period of watching the film clip also confirmed this. At the physiological level, there were no differences between the emotion experience of patients with schizophrenia and that of the healthy group, showing comparable brain activation (Kring and Barch, 2014). This confirmed the hypothesis that patients with schizophrenia experienced anger in a normal manner.

Even so, in the process of watching the film, the brain activation patterns of patients with schizophrenia and healthy participants showed significant differences on some channels at some time points. Differences in the process of emotion experience ultimately did not affect the subjective evaluation of emotion experience, and the subjective ratings of emotion experience were similar between the two groups. It may be that the act of watching the film, the constant stimulus of watching it, or the anger depicted in the film itself caused differences in activation or brain connections (Athanassiou et al., 2021) depending on how the two groups experienced the film. Since this study focused on the emotion expression aspect, differences in the process of watching the film will not be discussed in detail. However, these results suggest that there are still some unknown brain activities in the emotion experience of the two groups. In the future, experiments can be designed specifically to elucidate this aspect and explore the differences and their underlying causes.

### Brain Mechanisms Underlying Anger Expression Through Drawing

HbO concentrations during emotion expression showed that, compared to the healthy group, patients with schizophrenia showed significant negative activation in many areas of the PFC. This result confirmed that PFC damage affects emotion expression in schizophrenia. The task-induced decrease in brain activity is often called deactivation (Binder, 2012). In this study, blood oxygen concentrations, being lower in the task state than in the resting state, were taken to represent deactivation in the task stage.

In this study, patients with schizophrenia showed more deactivation of brain regions during the drawing process, which may be related to attentional bias. The PFC plays an important role in cognitive control (Miller and Cohen, 2001). Deactivation occurs when active control strategies are important, such as when encoding and actively maintaining cues, or when attentional bias and action preparation are required (Paxton et al., 2008). In this study, the deactivation of many brain regions in the PFC in both groups may be due to the need to maintain attention and suppress other unrelated cues while watching the movie clip and drawing. For patients with schizophrenia, a stronger attentional bias is required to reach the same level as normal individuals, which may cause a stronger deactivation.

In this study, the frontopolar area (BA10) included the frontal pole and part of the mPFC, which is part of the default mode network (DMN)—the brain network in a state of rest when no external task is being processed (Raichle et al., 2001). Most studies suggest that the DMN plays an important role in self-cognition and the detection of environmental attention (Mantini and Vanduffel, 2013; Li and Shu, 2014; Raichle, 2015). Patients with schizophrenia have an abnormal DMN (Abigail G. Garrity et al., 2007), including abnormalities or deactivation in various experimental tasks and excessive activation during the resting state (Broyd et al., 2009; Hu et al., 2017). Therefore, the significant deactivation of BA10-related channels in the experimental group during the emotion expression stage in this study may be due to the abnormal deactivation of the DMN in patients with schizophrenia caused by emotion expression tasks. The DMN is activated during the processing of self-related concepts. The deactivation in this study may be because the patients with schizophrenia did not process self-related information during emotion expression (Raichle et al., 2001; Buckner et al., 2008). The DMN is deactivated when external attention is needed. Therefore, the deactivation in the emotion expression stage in this experiment may reflect that the patients with schizophrenia concentrated their attention on the external world (Li and Shu, 2014)—i.e., they failed to immerse themselves in emotion expression and were still paying attention to stimuli in the external world.

The dorsolateral prefrontal cortex (DLPFC) is associated with a number of higher cognitive functions. It has been suggested that the DLPFC is involved in the cognitive process of monitoring or reporting an individual’s mental state, including spontaneous thoughts and emotion expressions (Castelli et al., 2000). Frith and Frith (1999) proposed that this region is involved in the external expression of the ego state. Drawing, as a projection technology, is a process of concretely expressing the inner state of the individual. This brain region is also involved in the suppression of negative emotions (Anticevic et al., 2010). Deactivation of the DLPFC may indicate that patients with schizophrenia have lower cognitive inhibition of anger, lower attention to anger, and no change in self-cognition due to social factors. Furthermore, cognitive deficits in schizophrenia may be associated with abnormal activation of the DLPFC (Davidson and Heinrichs, 2003; Barch and Ceaser, 2012). For example, DLPFC activation is decreased during the visuospatial working memory maintenance stage in individuals with schizophrenia (Jalbrzikowski et al., 2018) and during active cognitive control tasks (Lesh et al., 2013). In the drawing stage of emotion expression in this study, patients with schizophrenia may have used less active control and expressed their emotions more through drawing, resulting in the DLPFC showing a negative activation state. In addition, the low blood oxygen concentration in the DLPFC may also be because the drawing process is a process of visual stimulation requiring continuous attention, during which the concentration of HbO continues to decrease (Matsuda and Hiraki, 2006).

### Characteristics of Using Drawing to Express Emotion

#### The Characteristics, Similarities, and Differences Between the Schizophrenia and Healthy Groups Using Drawing to Express Emotion

##### Similarities and differences in drawing characteristics and drawing content between the two groups

Drawing features mainly include drawing layout, color characteristics, and characteristics of lines and strokes. The differences between the two groups were mainly reflected in the latter. Patients with schizophrenia scored lower on line-related measures (fluency, variability, and quality) than the control individuals. This may be related to the low kinesthetic/sensory function of patients with schizophrenia according to the ETC model. These results are consistent with those of previous studies: the line drawing performance of patients with schizophrenia is significantly worse than that of normal control groups (Blyler et al., 1997), and patients with schizophrenia drew shorter clock pointer lines as compared to the control group (Bozikas et al., 2004).

In terms of the drawing contents, the self-rated arousal and valence of the patients with schizophrenia after watching the film were not significantly different from those of the control group. However, the scores for the intensity of emotion expressed and clarity of the theme expressed through the drawings were significantly lower among the patients with schizophrenia compared to those of the control group. This is mainly due to the inability to effectively construct a visual image with intent and to organize the contents of the drawing into an organic whole that clearly reflects the intended theme. This result is also consistent with those from previous studies: the emotion expression and emotion experience of patients with schizophrenia are separated. They show fewer emotion behaviors, but their reported emotion experience is the same as or even higher than that of healthy people (Berenbaum and Oltmanns, 1992; Kring et al., 1993; Kring and Neale, 1996). This may be due to the fact that patients with schizophrenia have difficulty amplifying emotion expression behaviors, which is significantly associated with experiencing negative symptoms, especially apathy (Henry et al., 2007). In the clock drawing test, participants’ performance depends on complete attention, verbal receptivity, executive function (such as planning, organization, parallel processing, and self-health), and visual construction skills (Freedman et al., 1994; Rouleau et al., 1996; Royall et al., 1998). Schizophrenia, on the other hand, causes extensive and multifaceted impairment in many areas of neurocognitive function, including semantic memory, attention, and executive function (Heinrichs and Zakzanis, 1998; Meltzer and McGurk, 1999), as such influencing the expression of emotions through drawing.

##### Group differences in wording description

There were significant differences between the two groups at the cognitive/symbolic level of the ETC model. Comparing the two groups’ descriptions of the drawings showed that schizophrenia patients’ ability to associate the drawings with the social meaning conveyed was significantly different from that of the healthy participants. The three drawing coding indicators with the highest level of difference between the two groups (*d* > 2) were related to textual descriptions of the drawing: the relation between text and reality, the quality and symbolism of the drawing, and consistency between drawings and text. For these three codes related to cognition/symbolic hierarchy in the ETC model, patients with schizophrenia scored lower indexes. Symbol function involves the formation of intuition and self-directed concepts, symbolic representation, integrated thinking, symbolic expression, analysis (Kagin and Lusebrink, 1978; Lusebrink, 1990, 1991), links the external entity with the internal image, and can describe the totality of the individual (May, 1960). Compared to the healthy population, patients with schizophrenia exhibit a lack of a sense of reality, a certain degree of loss of self, confusion in self-cognition, and feelings of separation from external entities (American Psychiatric Association, 2013). They further exhibited an inability to connect the reality with the self or connect the text description with the reality of the self to form a symbolic title of quality for their drawings. This suggests that patients with schizophrenia have difficulty associating emotion expressions used in drawings with the social meanings they convey. In addition, because patients with schizophrenia have flights of thought and working memory defects, they cannot actively guide their behavior according to the target information in their working memory (Barch and Ceaser, 2012). This may explain why their drawings and words could not reach a high degree of unity.

In terms of the meaning of verbal descriptions, the performance of patients with schizophrenia was significantly worse than that of healthy groups with a large effect size (*d* = 1.71). This may be because the participants had fewer words, lacked detailed description, and could not form meaningful descriptions due to problems in executive function, which cause disintegration of language. In addition, anger has the emotional characteristics of narrowing the cognitive range and inhibiting irrelevant information, leading to impulsivity. Healthy people express emotions based on the social environment such that their emotions can adapt to social requirements, whereas patients with schizophrenia may face certain obstacles in this ability. Patients with schizophrenia express their original feelings in a more straightforward—and sometimes violent—manner, regardless of situational or appropriate language.

##### Group differences in creativity

The fourth level of the ETC model is creativity. This study found that patients with schizophrenia showed lower creativity in drawing and understanding its meaning. The patients with schizophrenia had lower scores than those in the control group in terms of line change, number of elements in the drawing, content richness, clarity of theme, meaningfulness of the text description, and the consistency between text description and the artist’s reality. Previous research on schizophrenia and creativity has been inconclusive. Some studies have found that people with schizophrenia are less creative than healthy groups (Eisenman, 1990), whereas others have reported the opposite result (Jena and Ramachandra, 1995; Kinney et al., 2001; Rubinstein, 2008). Some studies have also found both positive and negative correlations between schizophrenia and creativity (Son et al., 2015). Our results clearly support the findings of the first of the aforementioned studies.

Although there were significant differences in drawing features, drawing contents, and text expression between the schizophrenia and healthy groups, the effect size of differences in the former two features were smaller whereas that of differences in text expression was larger. This is not surprising, given that previous studies have reported language difficulties in patients with schizophrenia (Faber and Reichstein, 1981; Nicolson et al., 2000; Kuperberg and Caplan, 2003; Mitchell and Crow, 2005). Verbal creative expression is particularly challenging for people with schizophrenia when compared to other forms of expression. The DSM-5 defines speech disorder as one of the five major symptoms of schizophrenia, and it is one of the critical criteria for the diagnosis of schizophrenia (American Psychiatric Association, 2013).

Therefore, individuals with schizophrenia have fewer differences from healthy people when using drawings compared to when using words to describe and associate. This also supports the idea that using drawings can help people with schizophrenia express their emotions better.

#### Relationship Between Emotions Expressed Through Drawing and Brain Activation Data

There was a significant correlation between the drawing data and brain activation data when the two groups expressed emotions. In the healthy group, codes related to the number of colors, line variability, the number of drawing elements, richness of the drawing, clarity of the theme of the drawing, richness of the literal description, and the quality and meaningfulness of the literal description were significantly positively correlated with activation of the right Broca’s area (Ch2) in the drawing stage. The right Broca’s area is primarily responsible for language production and the understanding of action (Nishitani et al., 2005). Therefore, higher richness of literal description, quality and symbolism of the title, and line variability were positively correlated with the activation of Broca’s area, indicating that the higher the degree of activation, the better the performance. Line variability, pressure, drawing space, the meaningfulness and richness of the literal description, and the quality and symbolism of the title were significantly positively associated with the activation of the frontopolar area and a small part of the DLPFC. In other words, the higher the rating score, the stronger the activation in the frontopolar area and in a small part of the DLPFC. Additionally, the frontopolar area plays an important role in the collection of individual internal information (Christoff and Gabrieli, 2000). A meta-analysis also revealed that the mechanism of emotion control depends on the frontal pole (Koch et al., 2018). Therefore, the stronger the frontal pole activation, the better the score of objective indicators of the drawing, and better the ability to express emotions through the drawing. As mentioned earlier, the DLPFC is involved in emotion expression (Castelli et al., 2000) and the external expression of the ego state (Frith and Frith, 1999). In this study, behavioral data and brain activation data were used to further support this point.

In schizophrenia patients, tinge, pressure, line fluency, variability and quality, drawing space, the richness of the drawing, intensity of emotion expressed in the drawing, clarity of the theme of the drawing, richness and meaningfulness of the literal description, and relation between wording and the participant’s reality were negatively correlated with the activation of the frontopolar area and a part of the mPFC (Ch8, Ch9, Ch10, and Ch12). The PFC is deactivated when attentional bias and preparation for action are required (Paxton et al., 2008). Therefore, a higher level of deactivation indicates more attention given to the task and higher drawing rating scores. Line fluency, quality and variability, number of drawing elements, drawing space, and the richness of the drawing were significantly negatively correlated with activation in the left DLPFC and Broca’s area (Ch15). The DLPFC is linked to many higher cognitive processes. Higher levels of deactivation corresponded to better performance on objective indicators for the drawing. Lower DLPFC activation results from active control (Lesh et al., 2013) and corresponded to higher ratings for the drawing. However, the relationship between drawing expression and deactivation of the Broca’s area in patients with schizophrenia is not clear. Future studies can be carried out to further assess the relationship between drawing expression and negative activation of the Broca’s area in patients with schizophrenia.

### Limitations

This study has a limited sample size of 17 schizophrenia patients, and its conclusion may not be generalizable. Therefore, further studies should be conducted on a larger sample. Moreover, an affect grid scale was used to assess pleasure and arousal in a short period of time. The indices reflecting high levels of arousal and low levels of pleasure cannot be used to classify anger with fear. Further, we supposed that the film clip, which was used to successfully elicit anger in participants in other studies (Ding et al., 2014; Shi et al., 2015; Shi, 2017), could also be used to elicit anger in our study. Besides we did not set the subjective evaluation measurement of a calm state on the affect grid scale before the emotion experience stage. As such, the emotional state of the participants before watching the film clip was unknown. Therefore, it is unclear whether the experience of anger in participants was due to viewing the film clip or whether it was a pre-existing state of anger in schizophrenia. Although there was no significant difference in average brain activation, patients with schizophrenia showed significantly different activation of some channels compared to the healthy group when watching the film. This may indicate group differences in the process of emotion experience.

The possible effect of medication on negative symptoms (the emotional experience and expression process) (Artaloytia et al., 2006) and fNIRS (Chou et al., 2017) did not include in our consideration in this study. Further should take the medication effect into consideration to control this effect.

This study focused on the activation of the PFC. However, deficits in PFC function in schizophrenia and emotion-related brain regions exist mainly in subcortical structures. And the analysis process use classical method without consider some new methods (Lim et al., 2020). Therefore, future research should combine fMRI and fNIRS to study the brain activation of subcortical structures during emotion experience and expression. In addition, we can enlarge the sample to conduct difference analysis of brain data when drawing in two groups (drawing vertically vs. drawing horizontally), further measure the subjective feelings of emotions after drawing, and compare the influence of emotion expression via drawing on individuals.

## Conclusion

There were no differences between patients with schizophrenia and the healthy group in the experience of anger elicited by the film clip, behavioral performance, or overall physiological data. However, there were significant between-group differences in brain activation patterns on some channels at a specific time during the film-clip-watching process. When engaged in a drawing exercise to express anger, the brain activation patterns of patients with schizophrenia were significantly different from those of healthy participants. These differences were mainly reflected in the deactivation of the DLPFC and part of the frontal pole region. There were significant differences between patients with schizophrenia and the healthy group in drawing features, content, and ability to describe the content of the drawings. The effect sizes of the differences in the latter were greater than those of the former two. In terms of emotional expression, the drawing coding data and brain activation data were significantly correlated within the schizophrenia and healthy groups, but the correlation patterns in the two groups differed.

## Data Availability Statement

The raw data supporting the conclusions of this article will be made available by the authors, without undue reservation.

## Ethics Statement

The studies involving human participants were reviewed and approved by the University Committee on Human Research Protection of East China Normal University, Ethics Committee of Shanghai Changning District Mental Health Center. The patients/participants provided their written informed consent to participate in this study.

## Author Contributions

WY was responsible for the overall scheduling, research design, data analysis, and drawing coding system, and contributed to writing and reviewing the manuscript. WJ and LC were responsible for the assessment, grouping, liaison, and arrangement of patients with schizophrenia. CS was responsible for research design, experiment implementation, and data collection. YY analyzed the fNIRS data and contributed to writing. XY contributed to drawing coding, drawing-related data analysis, and writing. All authors contributed to the article and approved the submitted version.

## Conflict of Interest

The authors declare that the research was conducted in the absence of any commercial or financial relationships that could be construed as a potential conflict of interest.

## Publisher’s Note

All claims expressed in this article are solely those of the authors and do not necessarily represent those of their affiliated organizations, or those of the publisher, the editors and the reviewers. Any product that may be evaluated in this article, or claim that may be made by its manufacturer, is not guaranteed or endorsed by the publisher.

## Footnotes

1
http://www.alivelearn.net/xjview/

2
http://www.nitrc.org/projects/bnv/

## References

[B1] Aghevli M. A. Blanchard J. J. Horan W. P. (2003). The expression and experience of emotion in schizophrenia: a study of social interactions. *Psychiatry Res.* 119 261–270. 10.1016/S0165-1781(03)00133-1 12914897

[B2] Alpert M. Shaw R. J. Pouget E. R. Lim K. O. (2002). A comparison of clinical ratings with vocal acoustic measures of flat affect and alogia. *J. Psychiatr. Res.* 36 347–353. 10.1016/S0022-3956(02)00016-X 12127603

[B3] American Psychiatric Association (2013). “Schizophrenia spectrum and other psychotic disorders,” in *Diagnostic and Statistical Manual of Mental Disorders*, 5th Edn, ed. Arlington V. A. (Washington, DC: American Psychiatric Association). 10.1176/appi.books.9780890425596.dsm02

[B4] Anticevic A. Repovs G. Barch D. M. (2010). Resisting emotional interference: brain regions facilitating working memory performance during negative distraction. *Cogn. Affect. Behav. Neurosci.* 10 159–173. 10.3758/CABN.10.2.159 20498341 PMC3856369

[B5] Artaloytia J. F. Arango C. Lahti A. Sanz J. Pascual A. Cubero P. (2006). Negative signs and symptoms secondary to antipsychotics: a double-blind, randomized trial of a single dose of placebo, haloperidol, and risperidone in healthy volunteers. *Am. J. Psychiatry* 163 488–493. 10.1176/appi.ajp.163.3.488 16513871

[B6] Askim K. Knardahl S. (2021). The influence of affective state on subjective-report measurements: evidence from experimental manipulations of mood. *Front. Psychol.* 12:601083. 10.3389/fpsyg.2021.601083 33679520 PMC7930079

[B7] Athanassiou M. Dumais A. Iammatteo V. De Benedictis L. Dubreucq J.-L. Potvin S. (2021). The processing of angry faces in schizophrenia patients with a history of suicide: an fMRI study examining brain activity and connectivity. *Prog. Neuro Psychopharmacol. Biol. Psychiatry* 107:110253. 10.1016/j.pnpbp.2021.110253 33485961

[B8] Baker J. M. Liu N. Cui X. Vrticka P. Saggar M. Hosseini S. M. H. (2016). Sex differences in neural and behavioral signatures of cooperation revealed by fNIRS hyperscanning. *Sci. Rep.* 6:26492. 10.1038/srep26492 27270754 PMC4897646

[B9] Balconi M. Tirelli S. Frezza A. (2015). Event-related potentials (ERPs) and hemodynamic (functional near-infrared spectroscopy, fNIRS) as measures of schizophrenia deficits in emotional behavior. *Front. Psychol.* 6:1686. 10.3389/fpsyg.2015.01686 26579058 PMC4630975

[B10] Barazzone N. Davey G. C. L. (2009). Anger potentiates the reporting of threatening interpretations: an experimental study. *J. Anxiety Disord.* 23 489–495. 10.1016/j.janxdis.2008.10.007 19070989

[B11] Barch D. M. Ceaser A. (2012). Cognition in schizophrenia: core psychological and neural mechanisms. *Trends Cogn. Sci.* 16 27–34. 10.1016/j.tics.2011.11.015 22169777 PMC3860986

[B12] Berenbaum H. Oltmanns T. F. (1992). Emotional experience and expression in schizophrenia and depression. *J. Abnorm. Psychol.* 101 37–44. 10.1037//0021-843x.101.1.37 1537971 PMC4370316

[B13] Binder J. R. (2012). Task-induced deactivation and the “resting” state. *NeuroImage* 62 1086–1091. 10.1016/j.neuroimage.2011.09.026 21979380 PMC3389183

[B14] Blyler C. R. Maher B. A. Manschreck T. C. Fenton W. S. (1997). Line drawing as a possible measure of lateralized motor performance in schizophrenia. *Schizophr. Res.* 26, 15–23. 10.1016/s0920-9964(97)00040-6 9376334

[B15] Bozikas V. P. Kosmidis M. H. Gamvrula K. Hatzigeorgiadou M. Kourtis A. Karavatos A. (2004). Clock drawing test in patients with schizophrenia. *Psychiatry Res.* 121 229–238. 10.1016/j.psychres.2003.07.003 14675742

[B16] Brennan P. A. Mednick S. A. Hodgins S. (2000). Major mental disorders and criminal violence in a Danish birth cohort. *Arch. Gen. Psychiatry* 57 494–500. 10.1001/archpsyc.57.5.494 10807490

[B17] Broyd S. J. Demanuele C. Debener S. Helps S. K. James C. J. Sonuga-Barke E. J. S. (2009). Default-mode brain dysfunction in mental disorders: a systematic review. *Neurosci. Biobehav. Rev.* 33 279–296. 10.1016/j.neubiorev.2008.09.002 18824195

[B18] Buckner R. L. Andrews-Hanna J. R. Schacter D. L. (2008). The brain’s default network. *Ann. N. Y. Acad. Sci.* 1124 1–38. 10.1196/annals.1440.011 18400922

[B19] Carver C. S. (2004). Negative affects deriving from the behavioral approach system. *Emotion* 4 3–22. 10.1037/1528-3542.4.1.3 15053723

[B20] Castelli F. Happé F. Frith U. Frith C. (2000). Movement and mind: a functional imaging study of perception and interpretation of complex intentional movement patterns. *NeuroImage* 12 314–325. 10.1006/nimg.2000.0612 10944414

[B21] Chou P. H. Huang C. J. Sun C. W. (2020). The potential role of functional near-infrared spectroscopy as clinical biomarkers in schizophrenia. *Curr. Pharm. Des.* 26 201–217. 10.2174/1381612825666191014164511 31612819

[B22] Chou P. H. Lin W. H. Li W. R. Huang C. M. Sun C. W. (2017). Reduced language lateralization in first episode schizophrenia: a near infrared spectroscopy study. *Prog. Neuropsychopharmacol. Biol. Psychiatry* 78 96–104. 10.1016/j.pnpbp.2017.05.001 28499897

[B23] Christoff K. Gabrieli J. D. E. (2000). The frontopolar cortex and human cognition: evidence for a rostrocaudal hierarchical organization within the human prefrontal cortex. *Psychobiology* 28 168–186. 10.3758/BF03331976

[B24] Cohen A. S. Minor K. S. (2010). Emotional experience in patients with schizophrenia revisited: meta-analysis of laboratory studies. *Schizophr. Bull.* 36 143–150. 10.1093/schbul/sbn061 18562345 PMC2800132

[B25] Cui X. Bray S. Reiss A. L. (2010). Functional near infrared spectroscopy (NIRS) signal improvement based on negative correlation between oxygenated and deoxygenated hemoglobin dynamics. *NeuroImage* 49 3039–3046. 10.1016/j.neuroimage.2009.11.050 19945536 PMC2818571

[B26] Dalebroux A. Goldstein T. Winner E. (2008). Short-term mood repair through art-making: positive emotion is more effective than venting. *Motiv. Emot.* 32 288–295. 10.1007/s11031-008-9105-1

[B27] Davidson L. L. Heinrichs R. W. (2003). Quantification of frontal and temporal lobe brain-imaging findings in schizophrenia: a meta-analysis. *Psychiatry Res.* 122 69–87. 10.1016/s0925-4927(02)00118-x 12714172

[B28] Denzin N. K. (2017). *The Research Act: A Theoretical Introduction to Sociological Methods.* Piscataway, NJ: Transaction publishers.

[B29] Diliberto-Macaluso K. A. Stubblefield B. L. (2015). The use of painting for short-term mood and arousal improvement. *Psychol. Aesthet. Creat. Arts* 9 228–234. 10.1037/a0039237

[B30] Ding R. Wang F. Niu D. Li B. (2014). The effect of emotions associated with certainty (happiness, anger) and uncertainty (sadness) on trust (in Chinese: 高确定性情绪(开心、愤怒)与低确定性情绪(悲伤)对信任的影响). *J. Psychol. Sci.* 37 1092–1099.

[B31] Eisenman R. (1990). Creativity, preference for complexity, and physical and mental illness. *Creat. Res. J.* 3 231–236.

[B32] Faber R. Reichstein M. B. (1981). Language dysfunction in schizophrenia. *Br. J. Psychiatry* 139 519. 10.1192/bjp.139.6.519 7332856

[B33] Fassino S. Amianto F. Gastaldo L. Leombruni P. (2009). Anger and functioning amongst inpatients with schizophrenia or schizoaffective disorder living in a therapeutic community. *Psychiatry Clin. Neurosci.* 63 186–194. 10.1111/j.1440-1819.2009.01940.x 19335389

[B34] Feldman Barrett L. Russell J. A. (1998). Independence and bipolarity in the structure of current affect. *J. Pers. Soc. Psychol.* 74 967–984. 10.1037/0022-3514.74.4.967

[B35] Ferrari M. Quaresima V. (2012). A brief review on the history of human functional near-infrared spectroscopy (fNIRS) development and fields of application. *NeuroImage* 63 921–935. 10.1016/j.neuroimage.2012.03.049 22510258

[B36] Freedman M. Leach L. Kaplan E. Shulman K. Delis D. C. (1994). *Clock Drawing: A Neuropsychological Analysis.* Oxford: Oxford University Press.

[B37] Frith C. D. Frith U. (1999). Interacting minds–a biological basis. *Science* 286 1692–1695. 10.1126/science.286.5445.1692 10576727

[B38] Gaebel W. Wölwer W. (2004). Facial expressivity in the course of schizophrenia and depression. *Eur. Arch. Psychiatry Clin. Neurosci.* 254 335–342. 10.1007/s00406-004-0510-5 15365710

[B39] Garrity A. G. Pearlson G. D. McKiernan K. Lloyd D. Kiehl K. A. Calhoun V. D. (2007). Aberrant “default mode” functional connectivity in schizophrenia. *Am. J. Psychiatry* 164 450–457. 10.1176/ajp.2007.164.3.450 17329470

[B40] Gross J. J. Levenson R. W. (1995). Emotion elicitation using films. *Cogn. Emot.* 9 87–108. 10.1080/02699939508408966

[B41] Gühne U. Weinmann S. Arnold K. Ay E. S. Becker T. Riedel-Heller S. (2012). Künstlerische therapien bei schweren psychischen Störungen. *Nervenarzt* 83 855–860. 10.1007/s00115-011-3472-7 22733379

[B42] Guseva E. (2018). Bridging art therapy and neuroscience: emotional expression and communication in an individual with late-stage Alzheimer’s. *Art Ther.* 35 138–147. 10.1080/07421656.2018.1524260

[B43] Halberstadt A. G. Denham S. A. Dunsmore J. C. (2001). Affective social competence. *Soc. Dev.* 10 79–119. 10.1111/1467-9507.00150

[B44] Heinrichs R. W. Zakzanis K. K. (1998). Neurocognitive deficit in schizophrenia: a quantitative review of the evidence. *Neuropsychology* 12:426. 10.1037//0894-4105.12.3.426 9673998

[B45] Henry J. D. Green M. J. de Lucia A. Restuccia C. McDonald S. O’Donnell M. (2007). Emotion dysregulation in schizophrenia: reduced amplification of emotional expression is associated with emotional blunting. *Schizophr. Res.* 95 197–204. 17630254 10.1016/j.schres.2007.06.002

[B46] Hinz L. D. (2018). *Expressive Therapies Continuum: A Framework for Using Art in Therapy.* Taipei: Hung Yeh Publishing.

[B47] Hoshi Y. Kobayashi N. Tamura M. (2001). Interpretation of near-infrared spectroscopy signals: a study with a newly developed perfused rat brain model. *J. Appl. Physiol.* 90 1657–1662. 10.1152/jappl.2001.90.5.1657 11299252

[B48] Hu M.-L. Zong X.-F. Mann J. J. Zheng J.-J. Liao Y.-H. Li Z.-C. (2017). A review of the functional and anatomical default mode network in schizophrenia. *Neurosci. Bull.* 33 73–84. 10.1007/s12264-016-0090-1 27995564 PMC5567552

[B49] Izard C. (2007). Basic emotions, natural kinds, emotion schemas, and a new paradigm. *Perspect. Psychol. Sci.* 2:260. 10.1111/j.1745-6916.2007.00044.x 26151969

[B50] Jaeger S. R. Roigard C. M. Chheang S. L. (2021). The valence × arousal circumplex-inspired emotion questionnaire (CEQ): effect of response format and question layout. *Food Qual. Prefer.* 90:104172. 10.1016/j.foodqual.2020.104172

[B51] Jalbrzikowski M. Murty V. P. Stan P. L. Saifullan J. Simmonds D. Foran W. (2018). Differentiating between clinical and behavioral phenotypes in first-episode psychosis during maintenance of visuospatial working memory. *Schizophr. Res.* 197 357–364. 10.1016/j.schres.2017.11.012 29137828 PMC5948111

[B52] Jang K. Tak S. Jung J. Jang J. Jeong Y. Ye J. C. (2009). Wavelet minimum description length detrending for near-infrared spectroscopy. *J. Biomed. Opt.* 14:034004. 10.1117/1.3127204 19566297

[B53] Jena S. Ramachandra S. (1995). Creativity among schizophrenics and non-psychiatric individuals. *J. Pers. Clin. Stud.* 11 59–64.

[B54] Jiang J. Dai B. Peng D. Zhu C. Liu L. Lu C. (2012). Neural synchronization during face-to-face communication. *J. Neurosci.* 32 16064–16069. 10.1523/jneurosci.2926-12.2012 23136442 PMC6621612

[B55] Kagin S. L. Lusebrink V. B. (1978). The expressive therapies continuum. *Art Psychother.* 5 171–180. 10.1016/0090-9092(78)90031-5

[B56] Kaimal G. Ayaz H. Herres J. Dieterich-Hartwell R. Makwana B. Kaiser D. H. (2017). Functional near-infrared spectroscopy assessment of reward perception based on visual self-expression: coloring, doodling, and free drawing. *Arts Psychother.* 55 85–92. 10.1016/j.aip.2017.05.004

[B57] Kay S. R. Fiszbein A. Opler L. A. (1987). The positive and negative syndrome scale (PANSS) for schizophrenia. *Schizophr. Bull.* 13 261–276. 10.1093/schbul/13.2.261 3616518

[B58] Keltner D. Sauter D. Tracy J. Cowen A. (2019). Emotional expression: advances in basic emotion theory. *J. Nonverbal Behav.* 43 133–160. 10.1007/s10919-019-00293-3 31395997 PMC6687086

[B59] King J. Kaimal G. (2019). Approaches to research in art therapy using imaging technologies. *Front. Hum. Neurosci.* 13:159. 10.3389/fnhum.2019.00159 31156413 PMC6534043

[B60] Kinney D. K. Richards R. Lowing P. A. LeBlanc D. Zimbalist M. E. Harlan P. (2001). Creativity in offspring of schizophrenic and control parents: an adoption study. *Creat. Res. J.* 13 17–25.

[B61] Koch S. B. J. Mars R. B. Toni I. Roelofs K. (2018). Emotional control, reappraised. *Neurosci. Biobehav. Rev.* 95 528–534. 10.1016/j.neubiorev.2018.11.003 30412701

[B62] Kring A. M. Barch D. M. (2014). The motivation and pleasure dimension of negative symptoms: neural substrates and behavioral outputs. *Eur. Neuropsychopharmacol.* 24 725–736. 10.1016/j.euroneuro.2013.06.007 24461724 PMC4020953

[B63] Kring A. M. Caponigro J. M. (2010). Emotion in Schizophrenia. *Curr. Dir. Psychol. Sci.* 19 255–259. 10.1177/0963721410377599 22557707 PMC3340922

[B64] Kring A. M. Moran E. K. (2008). Emotional response deficits in schizophrenia: insights from affective science. *Schizophr. Bull.* 34 819–834. 10.1093/schbul/sbn071 18579556 PMC2632476

[B65] Kring A. M. Neale J. M. (1996). Do schizophrenic patients show a disjunctive relationship among expressive, experiential, and psychophysiological components of emotion? *J. Abnorm. Psychol.* 105:249. 10.1037//0021-843x.105.2.249 8723006

[B66] Kring A. M. Kerr S. L. Smith D. A. Neale J. M. (1993). Flat affect in schizophrenia does not reflect diminished subjective experience of emotion. *J. Abnorm. Psychol.* 102:507. 10.1037//0021-843x.102.4.507 8282918

[B67] Kumar V. Shivakumar V. Chhabra H. Bose A. Venkatasubramanian G. Gangadhar B. N. (2017). Functional near infra-red spectroscopy (fNIRS) in schizophrenia: a review. *Asian J. Psychiatry* 27 18–31. 10.1016/j.ajp.2017.02.009 28558892

[B68] Kuperberg G. Caplan D. (2003). “Language dysfunction in schizophrenia,” in *Neuropsychiatry*, eds Schiffer R. B. Rao S. M. Fogel B. S. (Philadelphia, PA: Lippincott Williams and Wilkins), 444–466.

[B69] Lee S.-Y. Kim M.-D. Seo J. S. Lee K. Jon D.-I. Bahk W.-M. (2019). The effects of group arts therapy based on emotion management training on the emotional expression, alexithymia, depression, and quality of life in patients with schizophrenia. *Schizophr. Bull.* 45(Suppl. 2):S243. 10.1093/schbul/sbz019.383 20421336

[B70] Lesh T. A. Westphal A. J. Niendam T. A. Yoon J. H. Minzenberg M. J. Ragland J. D. (2013). Proactive and reactive cognitive control and dorsolateral prefrontal cortex dysfunction in first episode schizophrenia. *Neuroimage Clin.* 2 590–599. 10.1016/j.nicl.2013.04.010 24179809 PMC3777717

[B71] Leucht S. Kane J. M. Kissling W. Hamann J. Etschel E. Engel R. R. (2005). What does the PANSS mean? *Schizophr. Res.* 79 231–238. 10.1016/j.schres.2005.04.008 15982856

[B72] Levin S. Hall J. A. Knight R. A. Alpert M. (1985). Verbal and nonverbal expression of affect in speech of schizophrenic and depressed patients. *J. Abnorm. Psychol.* 94 487–497. 10.1037//0021-843x.94.4.487 4078152

[B73] Li Y. Shu H. (2014). The brain mechanisms and functional hypothesis of default mode network and its clinical application (in Chinese: 默认网络的神经机制、功能假设及临床应用). *Adv. Psychol. Sci.* 22 234–249.

[B74] Lim L. G. Ung W. C. Chan Y. L. Lu C. K. Sutoko S. Funane T. (2020). A unified analytical framework with multiple fnirs features for mental workload assessment in the prefrontal cortex. *IEEE Trans. Neural Syst. Rehabil. Eng.* 28 2367–2376. 10.1109/TNSRE.2020.3026991 32986555

[B75] Lusebrink V. B. (1990). *Imagery and Visual Expression in Therapy.* New York, NY: Plenum Press.

[B76] Lusebrink V. B. (1991). A systems oriented approach to the expressive therapies: the expressive therapies continuum. *Arts Psychother*. 18 395–403.

[B77] Lusebrink V. B. Hinz L. D. (2020). Cognitive and symbolic aspects of art therapy and similarities with large scale brain networks. *Art Ther.* 37 113–122. 10.1080/07421656.2019.1691869

[B78] Ma H. Chen S. Fu C. Zheng H. Zhang C. Wu X. (2013). Relationship between psychological state and house—tree—person drawing characteristics of rehabilitation patients with schizophrenia. *Chin. Gen. Pract.* 16 2293–2295.

[B79] Mantini D. Vanduffel W. (2013). Emerging roles of the brain’s default network. *Neuroscientist* 19 76–87. 10.1177/1073858412446202 22785104

[B80] Matsuda G. Hiraki K. (2006). Sustained decrease in oxygenated hemoglobin during video games in the dorsal prefrontal cortex: a NIRS study of children. *NeuroImage* 29 706–711. 10.1016/j.neuroimage.2005.08.019 16230030

[B81] May R. (1960). The significance of symbols. *ETC Rev. Gen. Semant.* XVII, 301–338.

[B82] McDonald N. M. Perdue K. L. Eilbott J. Loyal J. Shic F. Pelphrey K. A. (2019). Infant brain responses to social sounds: a longitudinal functional near-infrared spectroscopy study. *Dev. Cogn. Neurosci.* 36:100638. 10.1016/j.dcn.2019.100638 30889544 PMC7033285

[B83] Meltzer H. Y. McGurk S. R. (1999). The effects of clozapine, risperidone, and olanzapine on cognitive function in schizophrenia. *Schizophr. Bull.* 25 233–256. 10416729 10.1093/oxfordjournals.schbul.a033376

[B84] Meng Z. (2005). *Emotion Psychology (in Chinese: 情绪心理学).* Beijing: Peking University Press.

[B85] Miller E. K. Cohen J. D. (2001). An integrative theory of prefrontal cortex function. *Annu. Rev. Neurosci.* 24 167–202. 10.1146/annurev.neuro.24.1.167 11283309

[B86] Mitchell R. L. Crow T. J. (2005). Right hemisphere language functions and schizophrenia: the forgotten hemisphere? *Brain* 128 963–978. 15743870 10.1093/brain/awh466

[B87] Nakano S. Shoji Y. Morita K. Igimi H. Sato M. Ishii Y. (2018). Comparison of changes in oxygenated hemoglobin during the tree-drawing task between patients with schizophrenia and healthy controls. *Neuropsychiatr. Dis. Treat.* 14 1071–1082. 10.2147/ndt.s159984 29719398 PMC5916263

[B88] Ni K. Zou Y.-M. Chan R. C. (2017). Pleasure experience, emotion expression and regulation in schizophrenia: a review (in Chinese: 精神分裂症患者的情绪体验、表达与调节). *Chin. Mental Health J.* 31 432–435.

[B89] Nicolson R. Lenane M. Singaracharlu S. Malaspina D. Giedd J. N. Hamburger S. D. (2000). Premorbid speech and language impairments in childhood-onset schizophrenia: association with risk factors. *Am. J. Psychiatry* 157 794–800. 10784474 10.1176/appi.ajp.157.5.794

[B90] Nishitani N. Schurmann M. Amunts K. Hari R. (2005). Broca’s region: from action to language. *Physiology* 20 60–69. 10.1152/physiol.00043.2004 15653841

[B91] Olderbak S. G. Geiger M. Hauser N. C. Mokros A. Wilhelm O. (2021). Emotion expression abilities and psychopathy. *Pers. Disord.* 10.1037/per0000444 [Epub ahead of print]. 33411561

[B92] Oorschot M. Lataster T. Thewissen V. Lardinois M. Wichers M. van Os J. (2013). Emotional experience in negative symptoms of schizophrenia–no evidence for a generalized hedonic deficit. *Schizophr. Bull.* 39 217–225. 10.1093/schbul/sbr137 22021660 PMC3523912

[B93] Paxton J. L. Barch D. M. Racine C. A. Braver T. S. (2008). Cognitive control, goal maintenance, and prefrontal function in healthy aging. *Cereb. Cortex* 18 1010–1028. 10.1093/cercor/bhm135 17804479 PMC2904686

[B94] Pinti P. Tachtsidis I. Hamilton A. Hirsch J. Aichelburg C. Gilbert S. (2020). The present and future use of functional near-infrared spectroscopy (fNIRS) for cognitive neuroscience. *Ann. N. Y. Acad. Sci.* 1464 5–29. 10.1111/nyas.13948 30085354 PMC6367070

[B95] Preacher K. J. Hayes A. F. (2008). Asymptotic and resampling strategies for assessing and comparing indirect effects in multiple mediator models. *Behav. Res. Methods* 40 879–891. 18697684 10.3758/brm.40.3.879

[B96] Qiu H.-Z. Ye Z.-J. Liang M.-Z. Huang Y.-Q. Liu W. Lu Z.-D. (2017). Effect of an art brut therapy program called go beyond the schizophrenia (GBTS) on prison inmates with schizophrenia in mainland China—a randomized, longitudinal, and controlled trial. *Clin. Psychol. Psychother.* 24 1069–1078. 10.1002/cpp.2069 28078741

[B97] Raichle M. E. (2015). The brain’s default mode network. *Annu. Rev. Neurosci.* 38 433–447. 10.1146/annurev-neuro-071013-014030 25938726

[B98] Raichle M. E. MacLeod A. M. Snyder A. Z. Powers W. J. Gusnard D. A. Shulman G. L. (2001). A default mode of brain function. *Proc. Natl. Acad. Sci. U.S.A.* 98 676–682. 10.1073/pnas.98.2.676 11209064 PMC14647

[B99] Reske M. Kellermann T. Habel U. Jon Shah N. Backes V. von Wilmsdorff M. (2007). Stability of emotional dysfunctions? A long-term fMRI study in first-episode schizophrenia. *J. Psychiatr. Res.* 41 918–927. 10.1016/j.jpsychires.2007.02.009 17467008

[B100] Reyna-Martinez M. Gonzalez-Romo R. A. Lopez J. A. (2019). Case study: art therapy care to primary caregiver, mother of a child with leukemia to promote emotional expression and functional coping. *Arteter. Pap. Arteter. Educ. Art. Incl. Soc.* 14 131–146. 10.5209/arte.65092

[B101] Rouleau I. Salmon D. P. Butters N. (1996). Longitudinal analysis of clock drawing in Alzheimer’s disease patients. *Brain Cogn.* 31 17–34. 8790932 10.1006/brcg.1996.0022

[B102] Royall D. R. Cordes J. A. Polk M. (1998). CLOX: an executive clock drawing task. *J. Neurol. Neurosurg. Psychiatry* 64 588–594. 9598672 10.1136/jnnp.64.5.588 PMC2170069

[B103] Rubinstein G. (2008). Are schizophrenic patients necessarily creative? A comparative study between three groups of psychiatric inpatients. *Pers. Individ. Dif.* 45 806–810.

[B104] Russell J. A. Barrett L. F. (1999). Core affect, prototypical emotional episodes, and other things called emotion: dissecting the elephant. *J. Pers. Soc. Psychol.* 76 805–819. 10.1037/0022-3514.76.5.805 10353204

[B105] Russell J. A. Weiss A. Mendelsohn G. A. (1989). Affect grid: a single-item scale of pleasure and arousal. *J. Pers. Soc. Psychol.* 57 493–502. 10.1037/0022-3514.57.3.493

[B106] Schultz S. H. North S. W. Shields C. G. (2007). Schizophrenia: a review. *Am. Fam. Physician* 75 1821–1829. 17619525

[B107] Shi T. (2017). *fNIRS Study on the Influence of Anger on Creative Cognitive Processes (in Chinese: 愤怒情绪影响创造性认知过程的fNIRS研究)* [Master thesis]. Xi’an: Shaanxi Normal University.

[B108] Shi Y. Xu F. Li Y. Liu C. (2015). The impact of specific negative emotions on the framing effect (in Chinese: 具体负性情绪对框架效应的影响). *Psychol. Res.* 8 18–25.

[B109] Shin J. Müller K.-R. Hwang H.-J. (2016). Near-infrared spectroscopy (NIRS)-based eyes-closed brain-computer interface (BCI) using prefrontal cortex activation due to mental arithmetic. *Sci. Rep.* 6:36203. 10.1038/srep36203 27824089 PMC5099935

[B110] Singh A. K. Dan I. (2006). Exploring the false discovery rate in multichannel NIRS. *NeuroImage* 33 542–549. 10.1016/j.neuroimage.2006.06.047 16959498

[B111] Small D. A. Lerner J. S. (2008). Emotional policy: personal sadness and anger shape judgments about a welfare case. *Polit. Psychol.* 29 149–168. 10.1111/j.1467-9221.2008.00621.x

[B112] Son S. Kubota M. Miyata J. Fukuyama H. Aso T. Urayama S.-I. (2015). Creativity and positive symptoms in schizophrenia revisited: structural connectivity analysis with diffusion tensor imaging. *Schizophr. Res.* 164 221–226. 25823399 10.1016/j.schres.2015.03.009

[B113] Strauss G. P. Herbener E. S. (2011). Patterns of emotional experience in schizophrenia: differences in emotional response to visual stimuli are associated with clinical presentation and functional outcome. *Schizophr. Res.* 128 117–123. 10.1016/j.schres.2011.01.010 21330110 PMC3085645

[B114] Stuss D. Gow C. Hetherington C. (1992). “No longer gage”: frontal lobe dysfunction and emotional changes. *J. Consult. Clin. Psychol.* 60 349–359. 10.1037//0022-006X.60.3.349 1619089

[B115] Tak S. Ye J. C. (2014). Statistical analysis of fNIRS data: a comprehensive review. *NeuroImage* 85 72–91. 10.1016/j.neuroimage.2013.06.016 23774396

[B116] Tong J. Yu W. Fan X. Sun X. Zhang J. Zhang J. (2021). Impact of group art therapy using traditional chinese materials on self-efficacy and social function for individuals diagnosed with schizophrenia. *Front. Psychol.* 11:571124. 10.3389/fpsyg.2020.571124 33551897 PMC7855174

[B117] Trémeau F. (2006). A review of emotion deficits in schizophrenia. *Dialogues Clin. Neurosci.* 8 59–70. 10.31887/DCNS.2006.8.1/ftremeau 16640115 PMC3181757

[B118] Van Os J. Kapur S. (2009). Schizophrenia. *Lancet* 374 635–645. 10.1016/S0140-6736(09)60995-8 19700006

[B119] van’t Wout M. Chang L. J. Sanfey A. G. (2010). The influence of emotion regulation on social interactive decision-making. *Emotion* 10 815–821. 10.1037/a0020069 21171756 PMC3057682

[B120] Volavka J. (1999). The neurobiology of violence. *J. Neuropsychiatry Clin. Neurosci.* 11 307–314. 10.1176/jnp.11.3.307 10440006

[B121] Xia M. Wang J. He Y. (2013). BrainNet viewer: a network visualization tool for human brain connectomics. *PLoS One* 8:e68910. 10.1371/journal.pone.0068910 23861951 PMC3701683

[B122] Yan C. Cao Y. Zhang Y. Song L.-L. Cheung E. F. C. Chan R. C. K. (2012). Trait and state positive emotional experience in schizophrenia: a meta-analysis. *PLoS One* 7:e40672. 10.1371/journal.pone.0040672 22815785 PMC3399884

[B123] Yan W. Zhang M. Liu Y. (2021). Regulatory effect of drawing on negative emotion: a functional near-infrared spectroscopy study. *Arts Psychother.* 74:101780. 10.1016/j.aip.2021.101780

[B124] Ye J. C. Tak S. Jang K. Jung J. Jang J. (2009). NIRS-SPM: statistical parametric mapping for near infrared spectroscopy. *NeuroImage* 44 428–447. 10.1117/12.762291 18848897

[B125] Zhang Y. Meng T. Hou Y. Pan Y. Hu Y. (2018). Interpersonal brain synchronization associated with working alliance during psychological counseling. *Psychiatry Res.* 282 103–109. 10.1016/j.pscychresns.2018.09.007 30292535

[B126] Zheng P. Liu C. Yu G. (2012). An overview of mood-induction methods (in Chinese: 情绪诱发方法述评). *Adv. Psychol. Sci.* 20 45–55.

[B127] Zhu Y. (2013). *Emotional Expression, Culture and Mental Health (in Chinese 情绪表达,文化与心理健康).* Tianjin: Nankai University Press.

