# Supplementary material for: Anger Experience and Anger Expression Through Drawing in Schizophrenia: An fNIRS Study
Source: Front Psychol. 2021 Sep 1;12:721148. doi: 10.3389/fpsyg.2021.721148 (PMC8441178; doi:10.3389/fpsyg.2021.721148)
Supplement: Supplementary file 1 [file Table_1.DOCX]

**Table 1**. Coding manual for drawings by participants

| Coding category | Subcategory | Operational definition | | Ratings |
| --- | --- | --- | --- | --- |
| Drawing Layout | Paper orientation | | The orientation of the drawing paper (horizontal vs. vertical) | 1=Horizontally 2=Vertically |
|  | Drawing space | | The area of the drawing occupied in the paper | 1=All filled  The minimum unit is 0.1. |
| Color characteristics | Tinge | | Whether the colors used in the drawing have a cool or warm tone | 1=All for the cool tone 5= All for the warm tone 3=The average |
|  | Number of colors | | The number of colors used in the drawing | Counting |
| Line and brushstroke characteristics | Line fluency | | The fluency of lines in the drawing | 1=Completely not smooth 5=Completely smooth |
|  | Line variability | | Whether the lines vary in length, slope, and direction | 1=The lines are rigid, with no changes in the length, slope, and direction 5=The lines are flexible and have variation |
|  | Line quality | | The stability and refinement of lines | 1=Very unstable and rough 5=Very stable and fine |
|  | Pressure | | Pressure of drawing stick on paper, heavy or light stroke | 1=Very light 5=Very heavy |
| Drawing contents | Number of drawing elements | | The number of things drawn on the paper | Recorded as counts. Things of the same kind are counted as 1, but if things of the same kind are painted with different colors or in different sizes, they are counted as different kinds. |
|  | Richness of the drawing | | The richness of the content of the drawing | 1=Very poor 5=Very rich |
|  | Intensity of the emotion expressed in drawings | | The intensity of the emotion conveyed by the drawing | 1=Very mild 5=Very strong 3=Neutral |
|  | Clarity of the theme of the drawing | | The distinct theme of the drawing connecting with the topic | 1=Not clear at all 5=Very specific and thematic orientation |
| Wording for the description of the drawing | Richness of literal description | | The completeness and richness of answers to the questions | 1=An incomplete description 5=Full and rich description |
|  | Meaningfulness of the literal description | | The meaningfulness of the literal description and relevance to the given topic | 1=Make no sense at all 5=Very meaningful |
|  | Relation between wording and the participant’s reality | | The degree of relation between the literal description and the participant’s reality | 1=Not connected at all 5=Highly connected |
|  | Quality and symbolism of the title | | The title the painter has chosen for the drawing is meaningful and symbolic | 1=No quality and symbolism 5= High quality and symbolism |
|  | Consistency between drawing and literal words | | The participant’s drawing and the literal description of it are consistent and echo each other. | 1=Low consistency 5=High consistency |

**Table 2**. Normality test of each index across the two groups

| Test category | *p* (K-S normal test) |
| --- | --- |
| △draw_ch1 | 0.007 |
| △draw_ch2 | 0.200 |
| △draw_ch3 | 0.200 |
| △draw_ch4 | 0.107 |
| △draw_ch5 | 0.200 |
| △draw_ch6 | 0.123 |
| △draw_ch7 | 0.000 |
| △draw_ch8 | 0.200 |
| △draw_ch9 | 0.028 |
| △draw_ch10 | 0.010 |
| △draw_ch11 | 0.200 |
| △draw_ch12 | 0.200 |
| △draw_ch13 | 0.004 |
| △draw_ch14 | 0.038 |
| △draw_ch15 | 0.166 |
| △draw_ch16 | 0.200 |
| arousal | 0.081 |
| pleasure | 0.017 |
| PANSS_total | 0.200 |
| PANSS_negative | 0.200 |
| PANSS_positive | 0.200 |
| General psychopathology | 0.136 |
| Course of the disease | 0.200 |
| Paper orientation | 0.000 |
| Tinge | 0.000 |
| Number of colors | 0.024 |
| Line fluency | 0.000 |
| Line variability | 0.039 |
| Line quality | 0.018 |
| Pressure | 0.000 |
| Number of drawing elements | 0.000 |
| Drawing space | 0.020 |
| Richness of the drawing | 0.046 |
| Intensity of the emotion expressed in drawings | 0.083 |
| Clarity of the theme of the drawing | 0.008 |
| Richness of literal description | 0.000 |
| Meaningfulness of the literal description | 0.010 |
| Relation between wording and the participant’s reality | 0.011 |
| Quality and symbolism of the title | 0.020 |
| Consistency between drawing and literal words | 0.000 |

**Table 3**. One-sample t-test results for the brain activation patterns in each channel during emotional experience in the two participant groups. ( ^*^ *p*<0.05, ^**^ *p*<0.01).

| Group | Channel | *t* | *p (fdr corrected)* |
| --- | --- | --- | --- |
| Schizophrenia  (*df* = 16) | Ch1 | -3.022 | .011^*^ |
|  | Ch2 | -3.647 | .007^**^ |
|  | Ch3 | -2.432 | .032^*^ |
|  | Ch4 | -4.207 | .003^**^ |
|  | Ch5 | -2.424 | .032^*^ |
|  | Ch6 | -5.278 | .000^**^ |
|  | Ch7 | -2.999 | .011^*^ |
|  | Ch8 | -3.276 | .008^**^ |
|  | Ch9 | -3.464 | .007^**^ |
|  | Ch10 | -5.202 | .000^**^ |
|  | Ch11 | -3.315 | .008^**^ |
|  | Ch12 | -3.571 | .007^**^ |
|  | Ch13 | -2.282 | .039^*^ |
|  | Ch14 | -5.390 | .000^**^ |
|  | Ch15 | -3.132 | .010^*^ |
|  | Ch16 | -1.423 | .174 |
| Healthy  (*df* = 17) | Ch1 | -1.297 | .242 |
|  | Ch2 | -4.138 | .011^*^ |
|  | Ch3 | -0.832 | .417 |
|  | Ch4 | -3.520 | .020^*^ |
|  | Ch5 | -3.298 | .020^*^ |
|  | Ch6 | -1.233 | .250 |
|  | Ch7 | -2.848 | .022^*^ |
|  | Ch8 | -2.092 | .075 |
|  | Ch9 | -2.984 | .022^*^ |
|  | Ch10 | -2.866 | .022^*^ |
|  | Ch11 | -2.375 | .047^*^ |
|  | Ch12 | -2.443 | .046^*^ |
|  | Ch13 | -3.163 | .020^*^ |
|  | Ch14 | -1.712 | .129 |
|  | Ch15 | -3.126 | .020^*^ |
|  | Ch16 | -1.803 | .119 |

**Table 4**. One-sample t-test (compared with 0) results for the brain activation patterns in each channel during emotional expression in the two participant groups. ( ^*^ *p*<0.05, ^**^ *p*<0.01).

| Group | Channel | *t* | *p (fdr corrected)* |
| --- | --- | --- | --- |
| Schizophrenia  (*df* = 16) | Ch1 | -2.621 | .030^*^ |
|  | Ch2 | -1.815 | .128 |
|  | Ch3 | -3.359 | .013^*^ |
|  | Ch4 | -3.091 | .019^*^ |
|  | Ch5 | -0.660 | .554 |
|  | Ch6 | -3.886 | .010^*^ |
|  | Ch7 | -1.186 | .289 |
|  | Ch8 | -3.358 | .013^*^ |
|  | Ch9 | -2.977 | .020^*^ |
|  | Ch10 | -2.793 | .023^*^ |
|  | Ch11 | -2.797 | .023^*^ |
|  | Ch12 | -3.579 | .013^*^ |
|  | Ch13 | -1.520 | .197 |
|  | Ch14 | -4.013 | .010^*^ |
|  | Ch15 | -1.474 | .197 |
|  | Ch16 | -0.107 | .916 |
| Healthy  (*df* = 17) | Ch1 | 1.792 | .485 |
|  | Ch2 | -0.555 | .782 |
|  | Ch3 | 1.150 | .624 |
|  | Ch4 | 0.392 | .861 |
|  | Ch5 | -0.960 | .624 |
|  | Ch6 | 1.305 | .624 |
|  | Ch7 | 0.981 | .624 |
|  | Ch8 | -1.326 | .624 |
|  | Ch9 | -0.751 | .740 |
|  | Ch10 | -0.279 | .885 |
|  | Ch11 | -1.820 | .485 |
|  | Ch12 | 0.219 | .885 |
|  | Ch13 | -0.604 | .782 |
|  | Ch14 | -0.082 | .936 |
|  | Ch15 | -1.018 | .624 |
|  | Ch16 | -2.836 | .182 |
